# New Mitochondria-Targeted Fisetin Derivative Compromises Mitophagy and Limits Survival of Drug-Induced Senescent Breast Cancer Cells

**DOI:** 10.1021/acs.jmedchem.4c01664

**Published:** 2024-09-25

**Authors:** Iwona Rzeszutek, Martyna Cybularczyk-Cecotka, Anna Deręgowska, Paulina Stec, Maciej Wnuk, Olga Kołodziej, Joanna Kałafut, Anna Wawruszak, Wojciech Witkowski, Grzegorz Litwinienko, Anna Lewińska

**Affiliations:** † Institute of Biotechnology, College of Natural Sciences, University of Rzeszow, Pigonia 1, 35-310 Rzeszow, Poland; ‡ Faculty of Chemistry, University of Warsaw, Pasteura 1, 02-093 Warsaw, Poland; § Department of Biochemistry and Molecular Biology, Medical University of Lublin, Chodźki 1, 20-093 Lublin, Poland

## Abstract

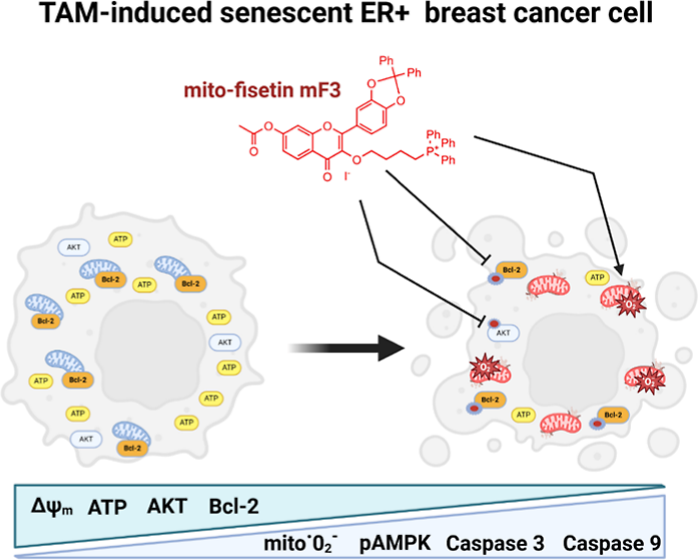

Mitochondria are considered as promising targets for cancer treatment. In the present study, triphenyl phosphonium cationic group-conjugated fisetin (mito-fisetin) was synthesized, and its anticancer activity was investigated in several cellular models of estrogen receptor (ER)-positive breast cancer in vitro and in vivo in proliferating and tamoxifen-promoted senescent states. Mito-fisetin, when used at low micromolar concentrations, stimulated the dissipation of mitochondrial membrane potential and oxidative stress, and affected mitochondrial function, resulting in apoptosis induction in senescent breast cancer cells. Mito-fisetin-mediated cytotoxicity was due to increased levels of phosphorylated AMPK, decreased levels of AKT and HSP90, and impaired mitophagic response, as judged by the analysis of the markers of mitophagosome formation. Senescent breast cancer cells were found to be more sensitive to mito-fisetin treatment than proliferating ones. We postulate that mitochondrial targeting in the case of fisetin may be considered as a promising anticancer and senotherapeutic strategy to eliminate drug-resistant senescent breast cancer cells.

## Introduction

Mitophagy, the selective elimination of damaged and/or old dysfunctional mitochondria by the involvement of classical autophagic machinery, is an evolutionally conserved mechanism to maintain mitochondrial homeostasis and cell survival.^1−3^ Furthermore, mitophagy may also be a regulator of mitochondrial quantity control by reducing mitochondrial mass as a part of adaptive response under conditions of energetic stress (e.g., hypoxia and nutrient starvation) to promote cellular fitness.^1−3^ Mitophagy may be executed by the means of several pathways relying or not relying on ubiquitin, for example, PTEN-induced putative kinase 1 (PINK1)/Parkin E3 ubiquitin ligase-mediated mitophagy^4−6^ and receptor-mediated mitophagy,^7^ respectively. Depolarization of mitochondrial membrane potential (MMP) may result in the stabilization of PINK1 at the outer mitochondrial membrane (OMM), PINK1-mediated phosphorylation of ubiquitin attached onto OMM proteins, the recruitment of Parkin and Parkin-mediated ubiquitination of OMM proteins leading to the recruitment of autophagy receptor proteins, and mitophagosome formation and subsequent mitochondrial autophagy.^2,3,8^ In contrast, for example, under hypoxic conditions, ubiquitin-independent mitophagy may be mediated by adaptor proteins containing mitochondrial-targeting domains and LC3-interacting region (LIR) motifs such as BNIP3 (Bcl-2/adenovirus E1B 19 kDa interacting protein 3), NIX (NIP-3-like protein X), FUNDC1 (FUN14 domain-containing protein 1), and Bcl-2L13 (Bcl-2-like 13, Bcl-rambo).^8−11^ Due to the fundamental role of mitochondria in energy metabolism, cell signaling, and cell death, limited mitochondrial functionality, as a result of aberrant mitophagic activity and diminished mitochondrial turnover, has been implicated in a number of pathophysiological conditions, including cancer and age-related diseases, and targeting mitophagy has been recognized as a novel therapeutic strategy.^2,12^

Fisetin (3,3′,4′,7-tetrahydroxy-flavone, 5-deoxyquercetin, F) is a naturally occurring flavonoid-based phytochemical with health-promoting effects due to its antioxidant, anti-inflammatory, antiaging, and anticancer properties.^13−15^ Fisetin may interfere with the activity of cell survival promoting signaling pathways such as PI3K/AKT/mTOR and mitochondrial function to stimulate anticancer effects by the inhibition of cell proliferation, metastatic potential, and angiogenesis and the induction of apoptotic cell death.^13−15^ Fisetin is also a promising senotherapeutic agent with potent senolytic activity against different types of senescent cells compared to other plant-derived substances such as quercetin, curcumin, and resveratrol.^16^ As fisetin is characterized by limited water solubility, stability, and bioavailability, several approaches, including nanobased approaches, have been proposed to augment its beneficial effects in biological systems.^15,17,18^ Conjugation with triphenyl phosphonium cationic (TPP^+^) groups can also be used to achieve 100- to 1000-fold accumulation of selected drugs and/or natural compounds in the mitochondrial compartment and induce anticancer effects by promoting mitochondrial dysfunction and related cytotoxicity.^19^ Therefore, we hypothesize that mitochondria-targeted triphenylphosphonium-based fisetin may exert more potent biological effects against cancer cells than unmodified fisetin in terms of mitochondria-focused anticancer action. To the best of our knowledge, mito-fisetin derivatives as TPP^+^-conjugates have never been obtained and analyzed as dysregulators of mitochondrial function in biological systems.

In the present study, two derivatives of mito-fisetin (mF3 and mF7) were synthesized and tested against four cellular models of estrogen receptor (ER)-positive breast cancer in vitro, namely, HCC1500, CAMA-1, HCC1428, and ZR-75-30 cells in proliferating and nonproliferating states, upon induction of senescence program by stimulation with the ER-targeting drug tamoxifen. The potent anticancer action of mito-fisetin mF3 compared to that of fisetin was documented with no effects against the normal corresponding cells. Senescent breast cancer cells were found to be more sensitive to mF3-associated mitochondria damaging effects than proliferating breast cancer cells, which facilitated their elimination due to impaired mitophagy and apoptosis induction. Anticancer effects of mF3 were also validated using an ER-positive breast cancer cell line-based xenograft system in vivo.

## Results and Discussion

### Mito-Fisetin mF3 Promotes Depolarization of MMP and Affects Mitochondrial Function in Drug-Induced Senescent Breast Cancer Cells

**Figure 1:**
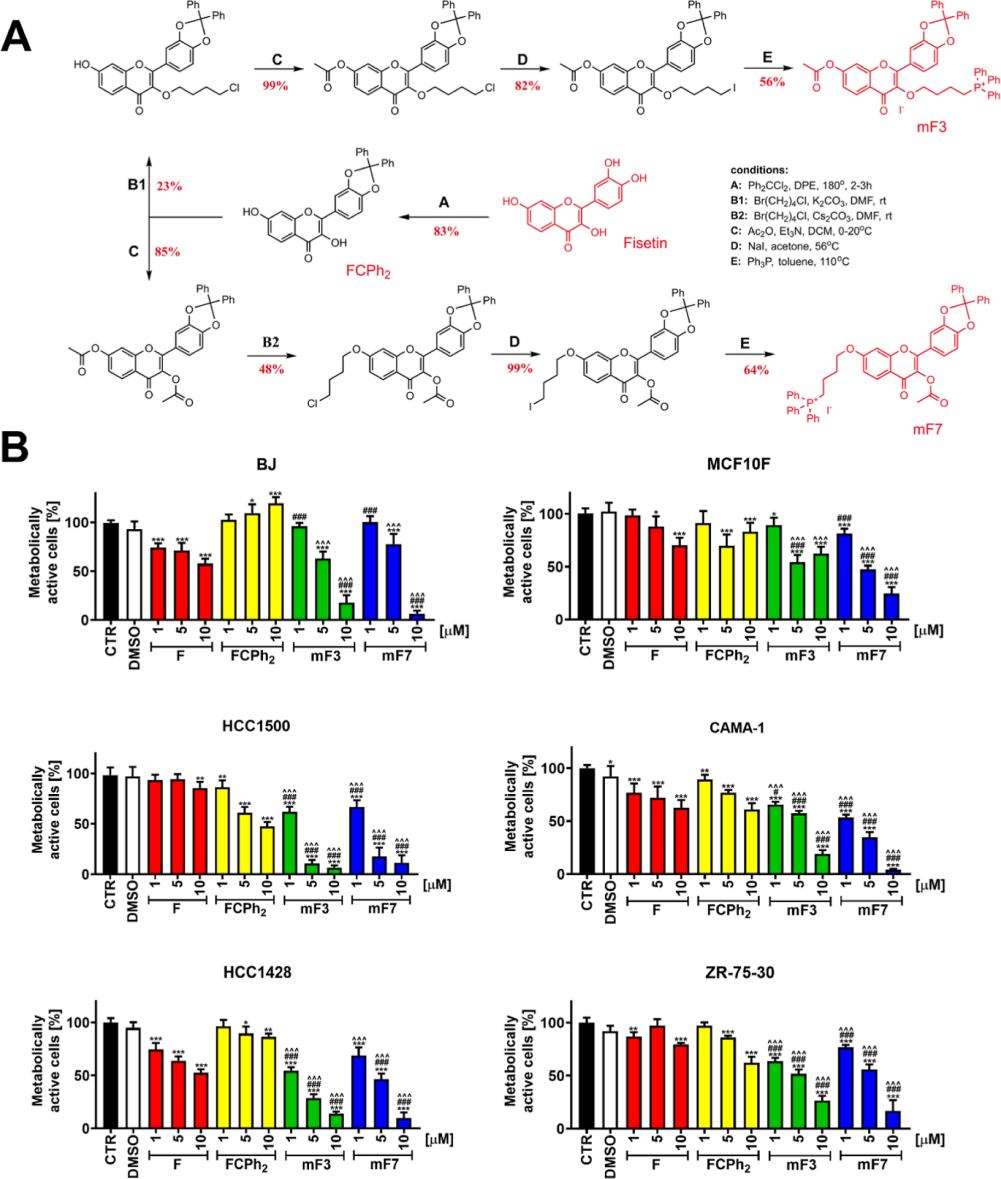
Strategy of transformation of fisetin into lipophilic derivatives with the *n*-butyltriphenylphosphonium cation attached at positions 3-O (mF3) or 7-O (mF7) and with other hydroxy groups blocked (e.g., FCPh_2_) (A). Numbers under the arrows indicate the yield of the reaction (in red, %). General conditions for reactions A–E are included. Detailed description of preparation and purification procedures for each step as well as for identification of each compound by ^1^H and ^13^C NMR is provided in the Supporting Information. (B) Changes in the metabolic activity of normal cells (BJ fibroblasts, noncancerous mammary epithelial MCF10F cells, and ER-positive breast cancer cells (HCC1500, CAMA-1, HCC1428, and ZR-75-30) upon stimulation with fisetin (F) and fisetin derivatives (FCPh_2_, mF3, and mF7). Cells were treated with fisetin and fisetin derivatives (1, 5, and 10 μM) for 24 h, and metabolic activity was assessed using MTT test. The effect of the solvent used (DMSO) is also presented. Bars indicate SD, *n* = 3, ***p < 0.001, **p < 0.01, *p < 0.05 compared to the corresponding untreated control (CTR) (ANOVA and Dunnett’s a posteriori test), ^###^*p* < 0.001, ^#^*p* < 0.05 compared to fisetin treatment (F) (ANOVA and Tukey’s a posteriori test), ^∧∧∧^*p* < 0.001 compared to FCPh_2_ treatment (ANOVA and Tukey’s a posteriori test).

The anticancer effects of three newly synthesized fisetin derivatives, namely, FCPh_2_ with blocked catechol moiety (3′,4′ position) and two other mitochondria-targeted FCPh_2_ with attached triphenyl phosphonium cationic (TPP^+^) group^19^ at position 3 or 7, mF3 and mF7, respectively (Figure 1A, synthetic details are denoted in Schemes S1–S11, NMR spectra of intermediate and final products are presented in Figures S1–S18, and HPLC profiles of final products are shown in Figure S19), were initially evaluated using MTT assay (Figure 1B). Due to the relatively low bioavailability of flavonoids, including fisetin, in biological systems,^13,15,17,18^ we have decided to study fisetin and fisetin derivative-mediated changes in metabolic activity up to the concentration of 10 μM (Figure 1B). The effects of two mito-fisetin derivatives mF3 and mF7 were the most pronounced against ER-positive breast cancer cells compared to fisetin (F) and FCPh_2_ stimulations. Normal cells were less sensitive to mito-fisetin treatment (Figure 1B). Some cell lines, namely, HCC1428 breast cancer cells and BJ human normal foreskin fibroblasts, were also sensitive to fisetin treatment, especially at the concentration of 10 μM (Figure 1B). As mF3-mediated changes in metabolic activity in three breast cancer cell lines were more pronounced compared to changes in metabolic activity promoted by the other mito-fisetin derivative mF7, mF3 mito-fisetin was selected for further analysis.

**Figure 2:**
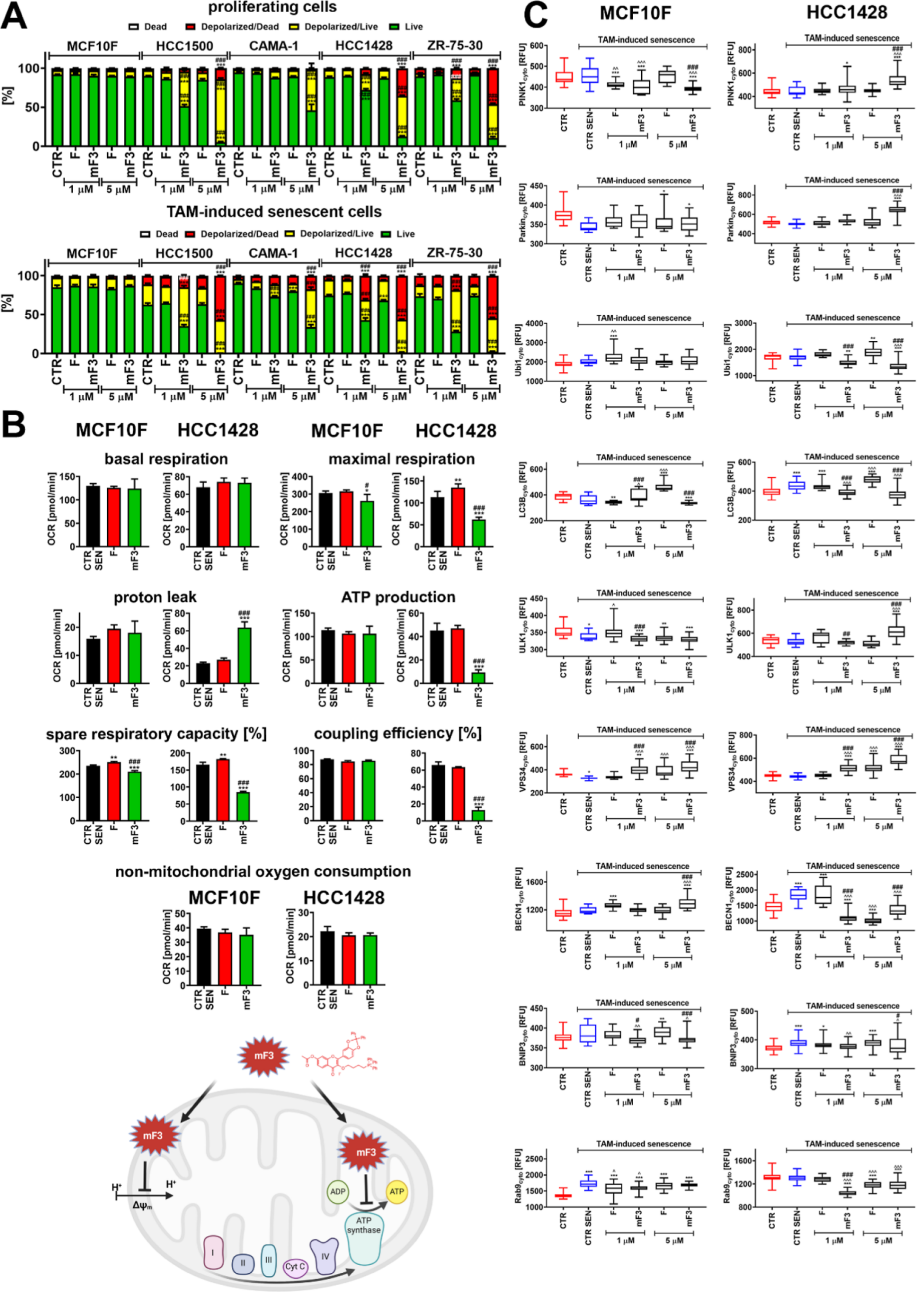
Mito-fisetin (mF3)-mediated changes in mitochondrial parameters (A,B) and mitophagic pathway (C) in tamoxifen-induced noncancerous and breast cancer cells. Senescent cells were treated with fisetin (F) and mito-fisetin (mF3) (1 or 5 μM) for 24 h (A,C) or 5 μM F or mF3 for 6 h (B). (A) Depolarization of the MMP (Δψ_m_) was assayed using a dedicated fluorogenic probe and flow cytometry. The effects in nonsenescent proliferating cells are also shown. (B) Mitochondrial function was assayed as real-time measurements of mitochondrial oxidative phosphorylation (OXPHOS) as selected OCR parameters (pmol/min or %), namely, basal respiration, ATP production, proton leak (stimulation with oligomycin), maximal respiration (stimulation with the uncoupler FCCP), and spare respiratory capacity (stimulation with rotenone and antimycin A). Uncoupling efficiency [%] and nonmitochondrial oxygen consumption [pmol/min] are also presented. A summarizing scheme showing mF3-mediated effects on mitochondrial function is also provided. (A,B) Bars indicate SD, *n* = 3, ***p < 0.001, **p < 0.01, *p < 0.05 compared to the corresponding untreated control (CTR, proliferating cells or CTR SEN, senescent cells) (ANOVA and Dunnett’s a posteriori test), ^###^*p* < 0.001, ^#^*p* < 0.05 compared to fisetin treatment (F) (ANOVA and Tukey’s a posteriori test). (C) Cytoplasmic levels of selected markers of mitophagy and autophagy (PINK1, Parkin, ubiquitin, LC3B, ULK1, VPS34, beclin 1, BNIP3, and Rab9) were studied using dedicated antibodies, an immunostaining protocol, and imaging cytometry. Protein levels are presented as relative fluorescent units (RFU). Box and whisker plots are shown, *n* = 3, ***p < 0.001, **p < 0.01, *p < 0.05 compared to the corresponding untreated proliferating control (CTR) (ANOVA and Dunnett’s a posteriori test), ^∧∧∧^*p* < 0.001, ^∧∧^*p* < 0.01, ^∧^*p* < 0.05 compared to the corresponding untreated senescent control (CTR SEN) (ANOVA and Dunnett’s a posteriori test), ^###^*p* < 0.001, ^##^*p* < 0.01, ^#^*p* < 0.05 compared to fisetin treatment (F) (ANOVA and Tukey’s a posteriori test).

mF3 caused depolarization of MMP in proliferating breast cancer cells when used at a concentration of 1 μM (Figure 2A). No effects on MMP were observed after fisetin treatment (Figure 2A). Surprisingly, mF3 did not affect MMP in normal MCF10F cells, even when used at a concentration of 5 μM (Figure 2A). At this higher concentration, cytotoxic effects were noticed in breast cancer cells as judged by an elevated fraction of depolarized dead cells, especially in HCC1428 and ZR-75-30 cells (Figure 2A). Fisetin has been previously proposed as a senotherapeutic agent.^13,16,17,20^ Therefore, we decided then to evaluate the anticancer effects of mito-fisetin mF3 also against ER-positive breast cancer cells at the senescent state.

To activate the senescence program, tamoxifen (TAM), a selective estrogen receptor modulator (SERM) widely used in hormonal therapeutics for hormone-sensitive breast cancer cells,^21^ was selected. Tamoxifen has been already reported to induce senescence in ER-positive breast cancer cells^22−24^ when used at low micromolar concentrations. For example, 10 μM tamoxifen promoted cellular senescence in ER-positive MCF-7 cells.^24^ In the present study, tamoxifen treatment conditions were optimized to stimulate drug-induced senescence in four cellular models of ER-positive breast cancer, and TAM-induced senescence was verified using selected markers of senescence, namely, senescence-associated beta-galactosidase activity and the levels of cell cycle inhibitor p21 (data not shown). The effect of different concentrations of tamoxifen on cell metabolic activity was also initially screened using MTT assay (Figure S20). TAM-induced senescent breast cancer cells were found to be more sensitive to changes in MMP than proliferating breast cancer cells (Figure 2A). Furthermore, elevated levels of depolarized dead cells were observed in mF3-treated TAM-induced senescent breast cancer cells compared to those in mF3-treated proliferating breast cancer cells (Figure 2A). MMP in mF3-treated TAM-induced senescent normal MCF10F cells was not affected (Figure 2A). One can conclude that the action of mF3 is selective to breast cancer cells, and drug-induced senescent breast cancer cells are more prone to mitochondria-targeted effects stimulated by mF3 than proliferating breast cancer cells. Thus, we have next focused more on TAM-induced senescent breast cancer cells in terms of mF3-mediated changes in the mitochondria (Figure 2B). We have considered real-time analysis of mitochondrial function by measuring the oxygen consumption rate (OCR) upon mF3 stimulation (Figure 2B).

Treatment with mF3 severely affected the selected mitochondrial parameters; e.g., mF3 promoted a decrease in maximal respiration, ATP production, spare respiratory capacity, and coupling efficiency and an increase in proton leak in TAM-induced senescent HCC1428 breast cancer cells (Figure 2B). Similar effects were not observed upon fisetin stimulation and in mF3-treated TAM-induced senescent normal MCF10F cells (Figure 2B). mF3 also affected mitochondrial function in proliferating breast cancer cells being ineffective against proliferating normal cells (Figure S21). The other mito-fisetin derivative mF7 stimulated similar changes in mitochondrial function in proliferating HCC1428 breast cancer cells compared to mF3 (Figure S21). In contrast, FCPh_2_, a fisetin derivative without the ability to accumulate in the mitochondria, exerted very slight effects on mitochondrial function in HCC1428 breast cancer cells (Figure S21). This suggests that mitochondrial targeting is essential to promote mito-stress upon stimulation with mF3 (Figure S21).

### Mito-Fisetin mF3 Compromises Mitophagy-Based Adaptive Response in Drug-Induced Senescent Breast Cancer Cells

As mitophagy, selective elimination of damaged or dysfunctional mitochondria, may be considered as an adaptive response to promote cell fitness and survival,^1−3,12^ we were then interested if mF3-mediated disruption of mitochondrial homeostasis (Figure 2A,B) may also stimulate mitophagy-based response in drug-induced breast cancer cells. As at least four pathways of mitophagy can be distinguished, such as ubiquitin-dependent mitophagy involving or not involving PINK1/Parkin, ubiquitin-independent mitophagy, or alternative mitophagy,^8^ we have analyzed selected parameters of different mitophagic responses upon stimulation with mF3 in TAM-induced senescent HCC1428 breast cancer cells (Figure 2C). Treatment with 5 μM mF3 resulted in an increase in the levels of PINK1 and Parkin, two important components of ubiquitin-dependent mitophagy,^4−6,8,25^ in drug-induced senescent breast cancer cells (Figure 2C). However, this was not accompanied by elevated levels of ubiquitin and microtubule-associated protein 1 light chain 3 beta (LC3B) (Figure 2C), a key factor for the formation of autophagosomes^26,27^ during mitophagy (so-called mitophagosomes). Thus, one can suggest that the mF3-mediated increase in the levels of PINK1 and Parkin is not a sufficient signal to induce mitophagosome formation and elimination of damaged mitochondria and promote fitness of mF3-treated breast cancer cells. On the other hand, two components of the classical autophagic machinery, also regulating different mitophagy pathways, namely, unc-51-like autophagy activating kinase 1 (ULK1), involved in the initiation step of autophagy as a part of ULK1 complex, and phosphoinositide-3-kinase class 3 (VPS34), involved in the phagophore formation,^8,26,27^ were also elevated in mF3-treated breast cancer cells (Figure 2C). As ULK1 can also be associated with ubiquitin-independent and alternative mitophagy,^8^ selected markers of ubiquitin-independent (i.e., BNIP3)^9,10^ and alternative (i.e., Rab9)^28,29^ mitophagy were also analyzed (Figure 2C). Mito-fisetin mF3 did not cause an increase in the levels of BNIP3 and Rab9 in drug-induced senescent breast cancer cells (Figure 2C). Mito-fisetin mF3 also did not promote an increase in the levels of beclin 1 (BECN1), a component of VPS34 complex^26,27^ (Figure 2C). Thus, perhaps mF3 is also not able to stimulate the turnover of damaged mitochondria by means of pathways other than PINK1/Parkin mitophagic pathways.

Data on fisetin-mediated mitophagy in cancer cells and related implications are scarce. For example, PINK1/Parkin-mediated mitophagy was reported in fisetin-treated pancreatic cancer cells as a response to mitochondrial stress;^30^ however, these effects were observed when fisetin was used at a very high concentration of 200 μM. In our experimental conditions, in contrast to mF3, fisetin at low micromolar concentrations did not affect MMP and mitochondrial function (Figure 2A,B). Thus, no induction of mitophagy can be expected as an adaptive response upon fisetin treatment in breast cancer cells (Figure 2C).

### Mito-Fisetin mF3 Induces Oxidative Stress in Mitochondria

**Figure 3:**
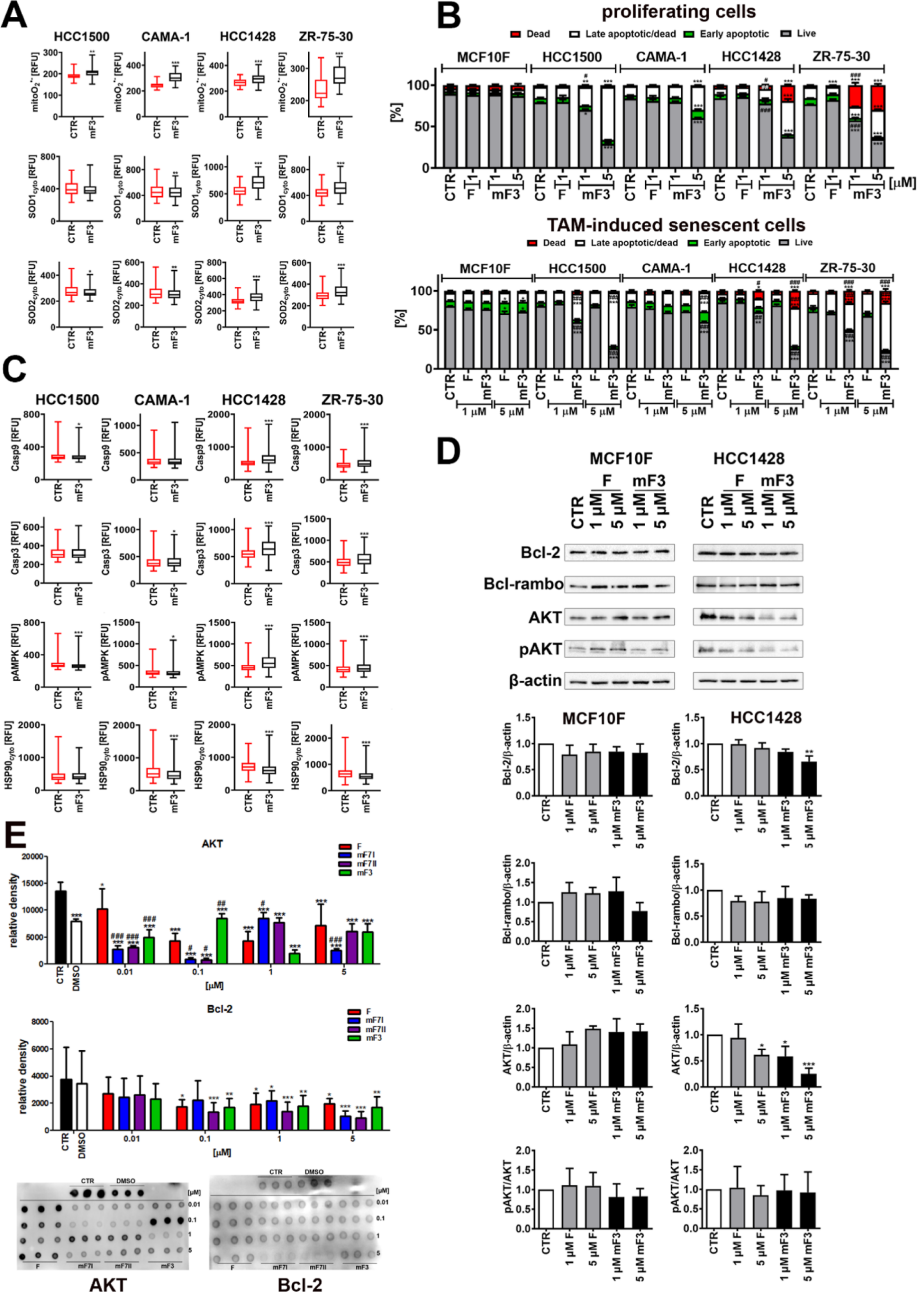
Mito-fisetin (mF3)-mediated oxidative stress (A), apoptosis and related parameters (B–D) in tamoxifen-induced senescent breast cancer cells, and interaction analysis of fisetin and fisetin derivatives with AKT and Bcl-2 in a cell-free system (E). Senescent cells were treated with fisetin (F) and mito-fisetin (mF3) (1 or 5 μM) for 24 h. (A) Selected parameters of oxidative stress and related responses were considered, namely, the levels of mitochondrial superoxide in live cells using a dedicated fluorogenic probe and imaging cytometry and the levels of superoxide dismutases cytosolic SOD1 and mitochondrial SOD2 in fixed cells using dedicated antibodies, an immunostaining protocol, and imaging cytometry. Results are presented as relative fluorescence units (RFU). Box and whisker plots are shown, *n* = 3, ***p < 0.001, **p < 0.01, *p < 0.05 compared to the corresponding untreated control (CTR) (Student’s *t*-test). (B) Phosphatidylserine externalization as a marker of apoptosis was analyzed by using Annexin V staining and flow cytometry. For comparison, the effects in proliferating breast cancer cells as well as normal proliferating and senescent MCF10F cells are also shown. Bars indicate SD, *n* = 3, ***p < 0.001, **p < 0.01, *p < 0.05 compared to the corresponding untreated control (CTR) (ANOVA and Dunnett’s a posteriori test), ^###^*p* < 0.001, ^##^*p* < 0.01, ^#^*p* < 0.05 compared to fisetin treatment (F) (ANOVA and Tukey’s a posteriori test). (C) Levels of caspase 9, caspase 3, phosphorylated AMPK, and HSP90 were investigated using dedicated antibodies, immunostaining protocols, and imaging cytometry. Results are presented as relative fluorescence units (RFU). Box and whisker plots are shown, *n* = 3, ***p < 0.001, *p < 0.05 compared to the corresponding untreated control (CTR) (Student’s *t*-test). (D) Western-blot-based analysis of the levels of Bcl-2, Bcl-rambo, AKT, and phospho-AKT. Data were normalized to a loading control (β-actin). For analysis of the phosphorylation status of AKT, the ratio of phospho-AKT to AKT was calculated. Bars indicate SD, *n* = 3, ***p < 0.001, **p < 0.01, *p < 0.05 compared to the corresponding untreated control (CTR) (ANOVA and Dunnett’s a posteriori test). Representative blots are also shown. (E) Interaction analysis between fisetin (F) or fisetin derivatives (mF3 or mF7, I—synthesis no. 1, II—synthesis no. 2) (0.01–5 μM) and AKT or Bcl-2 (50 ng) in a cell-free system upon incubation at 37 °C overnight using dot-blot analysis and dedicated antibodies. Results are presented as relative density units based on densitometry analysis. Representative dot-blots are presented. The effect of the solvent (DMSO) is also shown. Bars indicate SD, *n* = 3, ***p < 0.001, **p < 0.01, *p < 0.05 compared to the corresponding untreated control (CTR) (ANOVA and Dunnett’s a posteriori test), ^###^*p* < 0.001, ^##^*p* < 0.01, ^#^*p* < 0.05 compared to fisetin treatment (F) (ANOVA and Tukey’s a posteriori test).

As 5 μM mF3 promoted severe depolarization of MMP in drug-induced senescent breast cancer cells (Figure 2A), this concentration was selected for the analysis of the mF3-mediated redox imbalance and related response (Figure 3A). Treatment with mF3 resulted in an increase of mitochondrial superoxide production in TAM-induced senescent breast cancer cells that was accompanied by a superoxide dismutase (SOD)-mediated adaptive response (Figure 3A). The levels of cytosolic SOD1 and mitochondrial SOD2 were elevated in TAM-induced senescent HCC1428 and ZR-75-30 breast cancer cells (Figure 3A). Thus, oxidative stress may also be implicated in mF3-associated dissipation of MMP and mitochondrial dysfunction in senescent breast cancer cells.

### Mito-Fisetin mF3 Promotes Apoptotic Cell Death in Breast Cancer Cells

In general, fisetin has been reported to be active against different types of cancer cells at high concentrations that perhaps can question its usefulness as a chemotherapeutic agent or adjuvant taking into account its limited bioavailability.^13,14^ For example, fisetin treatment, at concentrations of 50 and 100 μM, resulted in apoptotic cell death in phenotypically different breast cancer cells in vitro, namely, ER-positive MCF-7 cells and triple-negative breast cancer (TNBC) cells.^31,32^ However, in our experimental settings, fisetin did not induce cytotoxic effects in proliferating or TAM-induced senescent breast cancer cells when used at concentrations of 1 or 5 μM (Figure 3B). In contrast, mF3 treatment resulted in the induction of apoptotic cell death in all four breast cancer cell lines at the same concentrations used, as judged by the analysis of phosphatidylserine externalization (Figure 3B). Apoptotic effects were potentiated when TAM-induced senescent HCC1500, HCC1428, and ZR-75-30 breast cancer cells were treated with mF3 (Figure 3B). Thus, senescent breast cancer cells were found to be more sensitive to mF3 treatment than proliferating breast cancer cells (Figure 3B). These results are in agreement with the observation that in mF3-treated drug-induced senescent breast cancer cells, the fraction of depolarized dead cells was increased compared to that of proliferating breast cancer cells treated with mF3 (Figure 2A). It is also important to note that proliferating normal cells were not sensitive to mF3-induced apoptosis, whereas mF3-mediated apoptotic effects were very slight in senescent normal cells compared to senescent breast cancer cells (Figures 3B and S22). In the case of mF3-treated drug-induced senescent HCC1428 and ZR-75-30 breast cancer cells, apoptosis induction was also accompanied by necrotic cell death (Figure 3B). This might suggest that other regulated modes of cell death may be involved in mF3-mediated cytotoxicity in breast cancer cells. However, mF3-induced necroptosis was ruled out as no increased phosphorylation of key necroptotic markers, namely, RIP1, RIP3, and MLKL,^33^ was revealed after mF3 treatment (Figure S23). Thus, MLKL cannot be oligomerized and translocated to the cell membrane to promote membrane rupture and necroptotic cell death^33^ in mF3-treated breast cancer cells. As senescent breast cancer cells were more prone to mF3-induced apoptosis than proliferating breast cancer cells, this mode of death was further analyzed in TAM-induced senescent breast cancer cells (Figure 3C). The involvement of caspase 9, an initiator caspase of the mitochondrial pathway of apoptosis, and caspase 3, a common executioner caspase of different apoptotic pathways, was documented in mF3-induced apoptosis in drug-induced senescent HCC1428 and ZR-75-30 breast cancer cells (Figure 3C). A decrease in the levels of antiapoptotic protein Bcl-2 was also observed in 5 μM mF3-treated senescent HCC1428 cells (Figure 3D). Similar effects were also noticed in proliferating cancer cells but not in normal proliferating and senescent MCF10F cells (Figures 3D and S24). However, no statistically significant changes in the levels of Bcl-rambo, a regulator of apoptosis^34^ and mitophagy,^11^ were revealed in mF3-treated senescent cells (Figure 3D). mF3-promoted cytotoxicity was also accompanied by increased phosphorylation of AMP-activated protein kinase (AMPK) in HCC1428 and ZR-75-30 breast cancer cells (Figure 3C), which may be due to the induction of mF3-associated energetic stress. Indeed, ATP production was decreased in mF3-treated HCC1428 breast cancer cells (Figure 2B). We have also previously shown that increased signals of phosphorylated AMPK mediated senolytic effects, namely, specific elimination of oxidant-induced senescent human fibroblasts upon stimulation with quercetin surface-functionalized Fe_3_O_4_ nanoparticles.^35^ Thus, the phosphorylation status of AMPK may contribute to senotherapeutic effects of different compounds in diverse cellular models of senescence (this study and ref (35)).

Furthermore, mF3-induced apoptosis in senescent breast cancer cells may also be mediated by decreased levels of heat shock protein 90 (HSP90) with antiapoptotic activity (Figure 3C). Molecular chaperones HSPs may confer resistance to apoptotic stimuli by different mechanisms, for example, by the interaction and stabilization of client proteins such as pro-survival kinase AKT.^36^ Thus, we have then analyzed if AKT levels may also be affected by mF3 treatment (Figure 3D). Indeed, a decrease in the pools of AKT was noticed in both mF3-treated proliferating and senescent HCC1428 breast cancer cells (Figures 3D and S24). Fisetin, when used at a concentration of 5 μM, also diminished the levels of AKT in senescent HCC1428 breast cancer cells (Figure 3D). We have also investigated the interaction between fisetin or mito-fisetin derivatives and AKT using a cell-free in vitro system and dot-blot assay (Figure 3E). Upon incubation with fisetin or mito-fisetin derivatives, chemiluminescence signals were decreased, which can suggest that fisetin and mito-fisetin derivatives may limit AKT stability and functionality. Similar effects were observed for the antiapoptotic protein Bcl-2 (Figure 3E). However, Bcl-2 is a mitochondrial protein, and one could speculate that the inhibitory effects of fisetin with no ability to enter the mitochondria would be minimal compared to mitochondria-targeted mF3 and mF7 in cellular systems. Of course, more studies are needed to document mito-fisetin-mediated changes in the stability and functionality of pro-survival factors such as AKT and Bcl-2 in the cells, especially since we have ruled out that mF3-mediated effects on AKT functionality may be mediated by decreased levels of phosphorylated AKT as judged by the unaffected ratio of phosphorylated AKT to AKT in mF3-treated breast cancer cells (Figure 3D).

**Figure 4:**
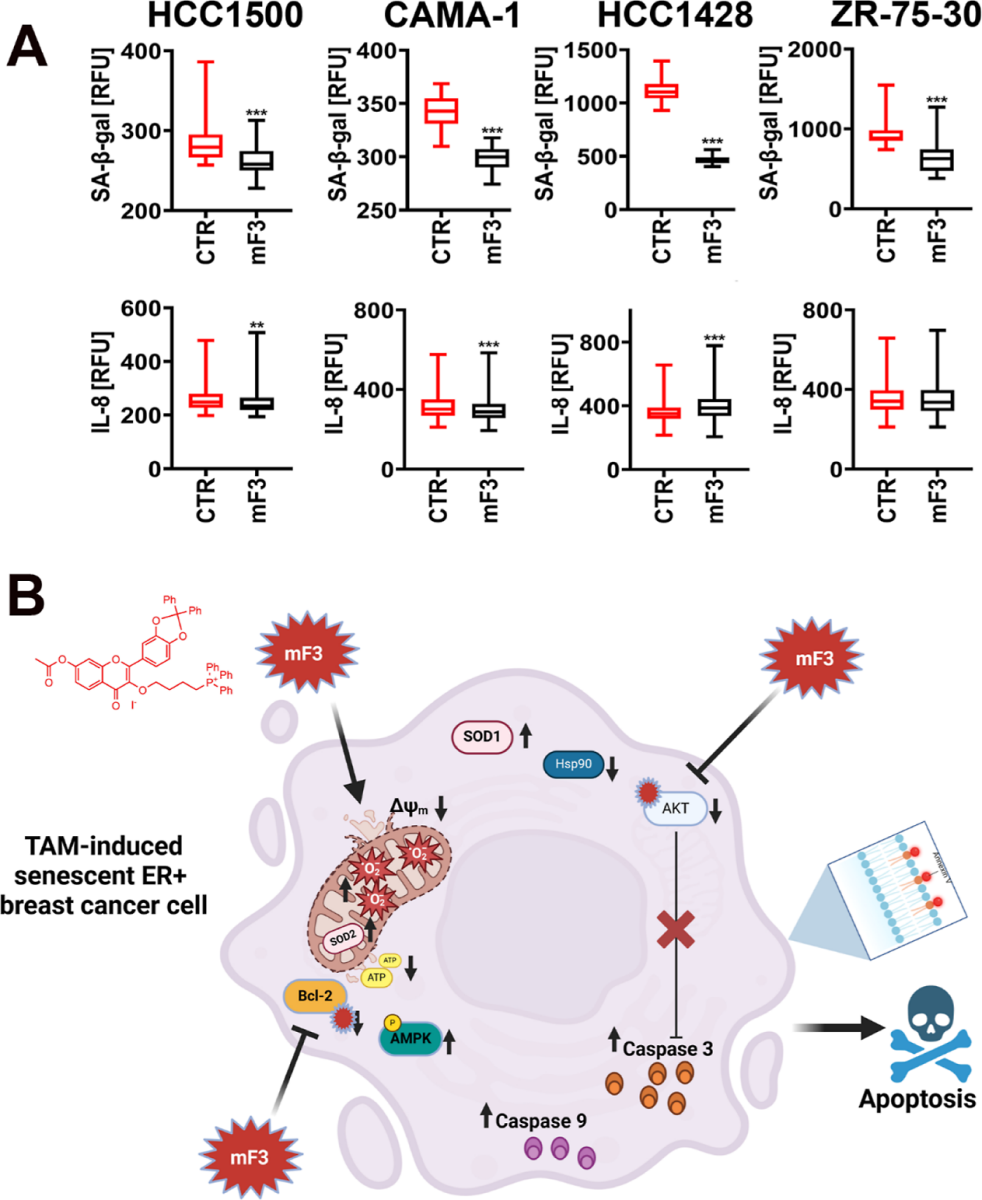
Mito-fisetin (mF3)-mediated changes in senescence parameters in tamoxifen-induced senescent breast cancer cells (A). Senescent cells were treated with 5 μM mF3 for 24 h. Senescence-associated β-galactosidase activity (SA-β-gal) was assayed using a dedicated staining protocol and imaging cytometry, whereas IL-8 levels were investigated using a dedicated antibody, an immunostaining protocol, and imaging cytometry. Results are presented as relative fluorescence units (RFU). Box and whisker plots are shown, *n* = 3, ***p < 0.001, **p < 0.01 compared to the corresponding untreated control (CTR) (Student’s *t*-test). (B) Summarizing scheme showing the effects of mito-fisetin (mF3) in tamoxifen-induced senescent breast cancer cells. Mito-fisetin promoted severe depolarization of mitochondrial membrane potential (Δψ_m_) and mitochondrial function leading to depletion of the ATP pool and induction of an AMPK-based response, oxidative stress, as judged by the increased production of mitochondrial superoxide, elevated levels of two superoxide dismutases, namely, cytosolic SOD1 and mitochondrial SOD2, and related cytotoxicity in senescent breast cancer cells. Mito-fisetin mF3 also decreased the levels of HSP90 and limited the functionality of pro-survival signals such as Bcl-2 and AKT, resulting in elevated levels of caspase 9 and caspase 3 and subsequent apoptotic cell death in senescent breast cancer cells. Thus, mitochondrial targeting approach in the case of fisetin promoted the elimination of drug-resistant senescent breast cancer cells.

Upon mF3 stimulation, the survivors of breast cancer cells were characterized by decreased senescence-beta-galactosidase activity (Figure 4A), a marker of cellular senescence.^37,38^ This result may suggest that mF3 has the ability to limit the senescent cell population upon activation of the senescence program by the treatment with the chemotherapeutic drug tamoxifen. The senescence-limiting effects were the most pronounced in mF3-treated HCC1428 cells (Figure 4A). Increased secretion of proinflammatory cytokines such as interleukin 8 (IL-8) is also another marker of senescence^39^ but may also be considered as an accompanying effect during different modes of cell death promoting immunogenic effects.^40,41^ Thus, the levels of IL-8 were also evaluated upon stimulation with mF3 (Figure 4A). However, IL-8 levels were decreased in HCC1500 and CAMA-1 cells, whereas the IL-8 pool was increased in HCC1428 cells after mF3 treatment. Thus, no clear effect of mF3 on cytokine secretion can be postulated, i.e., if mF3 can limit senescence-associated proinflammatory profiles or augment immunogenic response during cell death of necrotic morphotype.

The latter can also be ruled out as both mF3-treated HCC1428 and ZR-75-30 cells were characterized by an increased fraction of necrotic cell subpopulation (Figure 3B), but the levels of IL-8 were increased in HCC1428 cells and unaffected in ZR-75-30 cells after mF3 treatment (Figure 4A). Taken together, the mitochondrial targeting approach in the case of mito-fisetin mF3 resulted in severe depolarization of MMP, mitochondrial dysfunction, and oxidative stress, leading to apoptotic cell death in senescent breast cancer cells in vitro (Figure 4B). We showed that impaired mitophagic response may contribute to mF3-mediated cytotoxicity in senescent breast cancer cells.

### Mito-Fisetin mF3 Inhibits Tumor Size and Induces Cytotoxicity In Vivo

**Figure 5:**
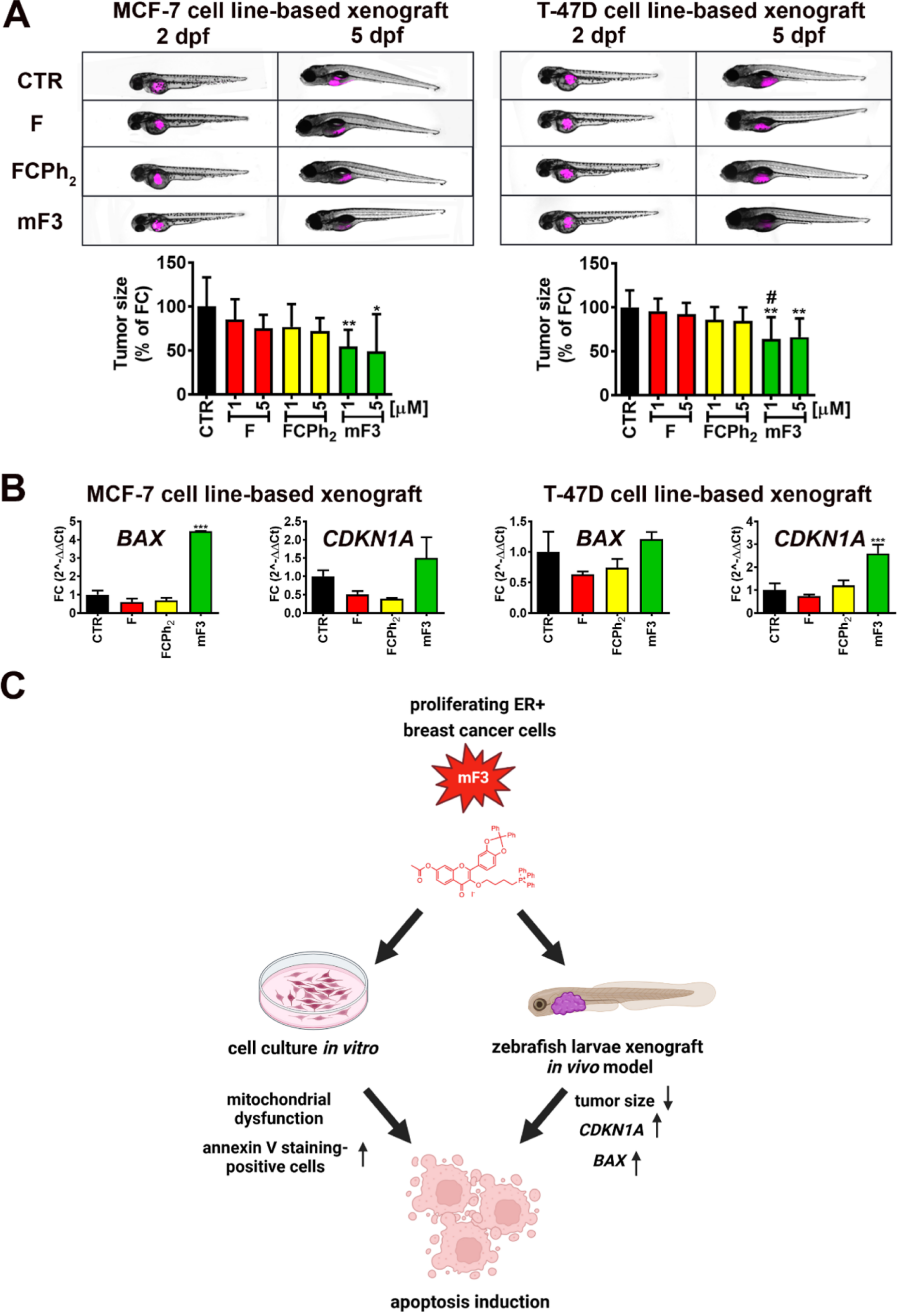
Anticancer effects of mito-fisetin (mF3) in vivo (A,B) and a summarizing scheme showing the effects of mito-fisetin (mF3) in proliferating breast cancer cells in vitro and in vivo (C). To assess antibreast cancer action of mF3 in vivo, the zebrafish (*Danio rerio*) model was used. Two ER-positive human breast cancer-based xenograft systems were considered, namely, MCF-7 and T-47D cell line-based xenograft models (A,B). Briefly, MCF-7 and T-47D cells were treated with fisetin (F) and fisetin derivatives (FCPh_2_ and mF3), labeled, and injected into the larval yolk cavity of zebrafish. 2 and 5 days postfertilization (dpf), animals were imaged and tumor size analysis was performed (A). Percentage of fold change (FC) in the tumor size is presented. Representative photographs are also shown upon stimulation with 5 μM fisetin or fisetin derivatives. (B) Real-time PCR-based analysis of mRNA levels of *BAX* and *CDKN1A* genes upon stimulation with 5 μM fisetin or fisetin derivatives. The levels of mRNA were calculated using delta CT subtraction and normalized. Bars indicate SD, *n* = 3, ***p < 0.001, **p < 0.01, *p < 0.05 compared to the corresponding untreated control (CTR) (ANOVA and Dunnett’s a posteriori test), ^#^*p* < 0.05 compared to fisetin treatment (F) (ANOVA and Tukey’s a posteriori test). (C) Summarizing scheme showing the anticancer effects of mito-fisetin (mF3) against proliferating ER-positive breast cancer cells in vitro (HCC1500, CAMA-1, HCC1428, and ZR-75-30 cell lines) and in vivo (MCF-7 cell line- and T-47D cell line-based xenografts using the zebrafish model). Mito-fisetin (mF3) promoted depolarization of the MMP and changes in mitochondrial function in breast cancer cells in vitro, induced cytotoxicity (apoptosis induction) both in vitro and in vivo, and inhibited tumor growth in vivo.

As we have documented, mF3 is also active against proliferating breast cancer cells in vitro in terms of mF3-mediated changes in metabolic activity, MMP and mitochondrial function, and apoptosis induction (Figures 1B, 2A, 3B, S21, and S24), we then decided to validate the antibreast cancer action of mF3 in vivo using the zebrafish (*Danio rerio*) model (Figure 5).

Two ER-positive human breast cancer-based xenograft systems were established using MCF-7 and T-47D cells, and antiproliferative effects of mF3 were shown as judged by decreased tumor size upon mF3 treatment. Similar effects were not observed upon stimulation with unmodified fisetin or fisetin derivative FCPh_2_ without attached triphenyl phosphonium cationic group (Figure 5A). Tumor inhibitory activity of mF3 was also accompanied by a statistically significant increase in mRNA levels of Bax proapoptotic protein in MCF-7 cell line-based xenografts and mRNA levels of p21, a cell cycle inhibitor in T-47D cell line-based xenografts (Figure 5B). Taken together, mito-fisetin mF3 is also active against proliferating breast cancer cells both in vitro and in vivo, and these anticancer effects are mediated by changes in MMP and mitochondrial function, apoptosis induction, and the inhibition of tumor growth (Figure 5C).

## Conclusions

Novel fisetin derivatives were synthesized and tested against ER-positive proliferating and senescent breast cancer cells using in vitro and in vivo models. We postulate that mito-fisetin mF3, at low micromolar concentrations, in contrast to unmodified fisetin, may be considered as a potent anticancer and senotherapeutic agent, especially for the elimination of drug-resistant senescent breast cancer cells.

## Experimental Section

### Synthesis of Mitochondria-Targeted Fisetin Derivatives

As fisetin is a polyhydroxyphenol with one enolic and three phenolic hydroxy groups, the preparation of derivatives with regioselectively attached butyl-triphenylphosphonium cation (with iodide counterion) had to be carried out in a multistep synthesis starting from protection of the catechol moiety (3′,4′ positions); see reaction A in Figure 1A with the resulting product 2-(2,2-diphenylbenzo[*d*][1,3]dioxol-5-yl)-3,7-dihydroxy-4*H*-chromen-4-one (FCPh_2_). After that, one synthesis pathway included direct (chloro)alkylation at position 7 (reaction B2) and subsequent acylation (process C) or, alternatively, a reversed sequence of acylation and chloroalkylation (reaction C followed by reaction B1, see Figure 1A). Then chlorine atom was replaced with iodine (reaction D), and finally, a terminal triphenylphosphonium cation was attached (reaction E), resulting in derivatives (4-((7-acetoxy-2-(2,2-diphenylbenzo[*d*][1,3]dioxol-5-yl)-4-oxo-4H-chromen-3-yl)oxy)butyl) triphenylphosphonium iodide (mF3) or (4-((3,5-diacetoxy-2-(2,2-diphenylbenzo[*d*][1,3]dioxol-5-yl)-4-oxo-4*H*-chromen-7-yl)oxy)butyl) triphenylphosphonium iodide (mF7). A similar methodology was applied by us previously for the preparation of quercetin with *n*-butyltriphenylphosphonium cation regioselectively substituted at position 3, 5, or 7.^42^ All of the reagents and solvents were purchased from commercial suppliers and used without further purification. NMR spectra were recorded at room temperature using a Bruker 300 MHz spectrometer unless otherwise specified. Detailed procedures and analytical techniques used for the identification of the products at each step of the synthesis are provided in Supporting Information (Schemes S1–S11; NMR spectra are also presented in Figure S1–S18). All fisetin derivatives are >99.9% pure by HPLC (Figure S19).

### ER-Positive Breast Cancer Cell Lines and Culture Conditions

Four cellular models of ER-positive breast cancer were used, namely, HCC1500 (CRL-2329, breast ductal carcinoma in situ), CAMA-1 (HTB-21, breast invasive lobular carcinoma), HCC1428 (CRL-2327, invasive breast carcinoma), and ZR-75-30 (CRL-1504, breast invasive lobular carcinoma also positive for HER2) (ATCC, Manassas, VA, USA). CAMA-1 cells were grown in Dulbecco’s modified Eagle medium (DMEM), and the remaining breast cancer cell lines were grown in Roswell Park Memorial Institute (RPMI) 1640 medium supplemented with 10% (v/v) fetal bovine serum (FBS) and antibiotic/antimycotic mix (100 U/ml penicillin, 0.1 mg/mL streptomycin, and 0.25 μg/mL amphotericin B) (Corning, Tewksbury, MA, USA). As control cells, a nontumorigenic epithelial cell line MCF10F (CRL-10318, ATCC, Manassas, VA, USA) was considered and cultured in DMEM/Ham’s Nutrient Mixture F12 (Merck KGaA, Darmstadt, Germany) containing 5% horse serum (H1270), 10 μg/mL human insulin, 10 ng/mL hEGF, 0.5 μg/mL hydrocortisone, 100 ng/mL cholera toxin from *Vibrio cholerae* (Merck KGaA, Darmstadt, Germany), and an antibiotic/antimycotic mix (100 U/mL penicillin, 0.1 mg/mL streptomycin, and 0.25 μg/mL amphotericin B) (Corning, Tewksbury, MA, USA). For the MTT assay, human normal proliferatively active fibroblasts (BJ, CRL-2522, ATCC, Manassas, VA, USA) were also used and cultured in DMEM with FBS and the antibiotic/antimycotic mix as stated above. All cell lines were routinely grown at 37 °C in a 5% CO_2_ incubator and passaged using a trypsin/EDTA solution.

### MTT Test

The effects of fisetin derivatives (FCPh_2_, mF3, mF7) were initially screened using the MTT assay. Briefly, breast cancer and normal cells (10^4^ cells per well of a 96-well plate) were treated with FCPh_2_, mF3, and mF7 at concentrations of 1, 5, and 10 μM for 24 h. Fisetin (F) at the same concentrations was used for comparison. The effects of solvent used (DMSO) were also considered. Mito-fisetin (mF3) was selected for further analysis based on the most pronounced changes in the metabolic activity of breast cancer cells.

### Cellular Model of Chemotherapy-Induced Senescence

Tamoxifen (TAM, T5648, Merck KGaA, Darmstadt, Germany) was used to activate the senescence program in ER-positive breast cancer and noncancerous cells. Briefly, cells were treated with 5 μM TAM for 24 h, and the drug was discarded and cells were left to grow for 48 h. Cells were then stimulated with 2.5 μM TAM for 24 h, and the drug was discarded and cells were left to grow for another 48 h. Cells were then again treated with 5 μM TAM for 6 h, and the drug was discarded and cells were left to grow for another 48 h. The induction of the senescence program was confirmed using selected markers of senescence (data not shown). TAM-induced senescent cells were then treated with 1 or 5 μM mF3 for 24 h to analyze the mF3-mediated effects on mitochondrial parameters, mitophagy, and cell death.

### Mitochondrial Membrane Potential Analysis

The effects of mF3 on mitochondrial membrane potential in proliferating and TAM-induced senescent cells were investigated using a Muse Cell Analyzer and a Muse Mitopotential Assay Kit (Cytek Biosciences, Amsterdam, The Netherlands), according to the manufacturer’s instructions. Upon dedicated staining and cytometric analysis, four cellular subpopulations were distinguished, namely, (1) cells with intact mitochondrial membrane and 7-AAD (−) (live cells), (2) cells with depolarized mitochondrial membrane and 7-AAD (−) (depolarized/live cells), (3) cells with depolarized mitochondrial membrane and 7-AAD (+) (depolarized/dead cells), and (4) cells with intact mitochondrial membrane and 7-AAD (+) (dead cells) (%).

### Real-Time Analysis of Mitochondrial Function

Proliferating and TAM-induced senescent cells were treated with 5 μM mF3 for 6 h and the selected mitochondrial parameters (basal respiration, maximal respiration, proton leak, ATP production, spare respiratory capacity, coupling efficiency, and nonmitochondrial oxygen consumption) were studied using a Seahorse XFp Analyzer (Seahorse Bioscience, Billerica, USA) and a Seahorse XFp Cell Mito Stress Test Kit (103010-100, Agilent Technologies, Santa Clara, CA, USA). For OCR analysis, oligomycin, carbonyl cyanide 4-(trifluoromethoxy) phenylhydrazone (FCCP), rotenone, and antimycin A were used according to the manufacturer’s instructions.^35^

### Imaging Cytometry

Upon treatment with mF3, TAM-induced senescent cells were fixed and immunostained^43^ to analyze the selected parameters of mitophagy, oxidative stress response, and cell death. The following primary and secondary antibodies were used, namely, anti-PINK1 (PA5-86941, 1:200), anti-Parkin (PA5–13399, 1:100), anti-Ubi1 (13–1600, 1:100), anti-LC3B (PA5–32254, 1:500), anti-ULK1 (20986–1-AP, 1:100), anti-BECN1 (TA502527, 1:100), anti-VPS34 (38-2100, 1:100), anti-BNIP3 (710728, 1:250), anti-Rab9 (MA3-067, 1:200), anti-SOD1 (PA1–30195, 1:200), anti-SOD2 (MA1-106, 1:200), anticaspase 3 (PA5-77887, 1:100), anticaspase 9 (PA5-17913, 1:100), antiphospho-AMPK alpha-1,2 (Thr183, Thr172) (PA5-17831, 1:200), anti-HSP90 (MA-110373, 1:200), anti-IL-8 (M801, 1:500), antimouse IgG conjugated to Texas Red-X (T-6390, 1:1000), antirabbit IgG conjugated to PE-cyanine5.5 (L43018, 1:1000), and antimouse IgG conjugated to FITC (F2761, 1:1000) (Thermo Fisher Scientific, Waltham, MA, USA). Nuclei were revealed by using Hoechst 33342 staining. The confocal imaging system IN Cell Analyzer 6500 HS and IN Carta software (Cytiva, Marlborough, MA, USA) were used to investigate fluorescence-signal-based protein levels. The protein levels are presented as relative fluorescence units (RFU).

### Western Blotting

Upon treatment with mF3, whole protein extracts were obtained from MCF10F and HCC1428 cells and the Western blotting protocol was applied as previously described.^35^ The following primary and secondary antibodies were used, namely, anti-RIP1 (3493, 1:1000), antiphospho-RIP1 (Ser166) (65746, 1:1000), anti-RIP3 (13526, 1:1000), antiphospho-RIP3 (Ser227) (93654, 1:1000), anti-MLKL (14993, 1:1000), antiphospho-MLKL (Ser358) (91689, 1:1000), anti-AKT (4691, 1:1000), antiphospho-AKT (Ser473) (44–621G, 1:1000), anti-Bcl-2 (MA5–41059, 1:1000), anti-Bcl-2L13 (Bcl-rambo) (16612–1-AP, 1:1000), anti-β-actin-peroxidase (A3854, 1:40000), antimouse IgG HRP-linked antibody (7076, 1:3000), and antirabbit IgG HRP-linked antibody (7074, 1:3000) (Thermo Fisher Scientific, Waltham, MA, USA, Merck KGaA, Darmstadt, Germany, and Cell Signaling Technology, Danvers, MA, USA). Protein levels were normalized to those of β-actin (loading control). Furthermore, mF3-mediated changes in the phosphorylation status of RIP1, RIP3, MLKL, and AKT were investigated as the ratios of phospho-RIP1 to RIP1, phospho-RIP3 to RIP3, phospho-MLKL to MLKL, and phospho-AKT to AKT, respectively.

### Mitochondrial Superoxide Analysis

Upon treatment with mF3, the levels of mitochondrial superoxide (mitoO_2_^·–^) in TAM-induced senescent cells were studied using MitoSOX Red superoxide indicator (Thermo Fisher Scientific, Waltham, MA, USA). Fluorescence signals were analyzed in live cells using a confocal imaging system, IN Cell Analyzer 6500 HS and IN Carta software (Cytiva, Marlborough, MA, USA). The levels of mitochondrial superoxide are presented as RFU.

### Senescence-Associated Beta–Galactosidase Activity Analysis

Upon treatment with mF3, senescence-associated beta–galactosidase activity in TAM-induced senescent cells was studied using a CellEvent Senescence Green Detection Kit (Thermo Fisher Scientific, Waltham, MA, USA) and a confocal imaging system IN Cell Analyzer 6500 HS and IN Carta software (Cytiva, Marlborough, MA, USA).^43^ Senescence-associated beta–galactosidase activity is presented as RFU.

### Annexin V Staining

Upon treatment with mF3, the phosphatidylserine externalization (a marker of apoptotic cell death) in proliferating and TAM-induced senescent cells was evaluated using flow cytometry and a Muse Annexin V & Dead Cell Kit (Cytek Biosciences, Amsterdam, The Netherlands), according to the manufacturer’s instructions. Briefly, four cell subpopulations were revealed (%), namely, (1) live cells negative for Annexin V staining and negative for 7-AAD staining, (2) early apoptotic cells positive for Annexin V staining and negative for 7-AAD staining, (3), late apoptotic cells/dead cells positive for Annexin V staining and positive for 7-AAD staining, and (4) dead (necrotic) cells negative for Annexin V staining and positive for 7-AAD staining.

### Interaction Analysis between Mito-Fisetin and Pro-survival Factors AKT and Bcl-2

The interaction between mito-fisetin derivatives and AKT or Bcl-2 was studied using a cell-free in vitro system. Briefly, 50 ng of recombinant human AKT/PKBα protein (14-276) or recombinant human Bcl-2 protein (SRP5340) (Merck KGaA, Darmstadt, Germany) was incubated with DMSO or different concentrations of fisetin or fisetin derivatives in a range between 0.01 and 5 μM in 20 mM Tris–HCl buffer (pH 8) containing 150 mM NaCl and 1 mM DTT at 37 °C overnight. Then, 2 μL of the samples was pipetted into an Amersham Protran nitrocellulose membrane (10600003, Merck KGaA, Darmstadt, Germany) and incubated with primary antibodies, namely, anti-AKT (4691, 1:3000, Cell Signaling Technology, Danvers, MA, USA) or anti-Bcl-2 (MA5–41059, 1:3000, Thermo Fisher Scientific, Waltham, MA, USA) at 4 °C overnight. The membranes were then washed 3x with TBS-T for 5 min and incubated with secondary antibodies antirabbit IgG (7074, 1:3000) or antimouse IgG (7076, 1:3000) (Cell Signaling Technology, Danvers, MA, USA) at RT for 1 h. Next, the membranes were washed 3x with TBS-T for 5 min and incubated with Clarity Western ECL substrate chemiluminescent detection reagent (Bio-Rad, Hercules, CA, USA) for 5 min prior to image acquisition (ChemiDoc imaging system, Bio-Rad, Hercules, CA, USA). Densitometry analysis of the dots was performed using ImageJ software (https://imagej.nih.gov/ij/). Results are presented as relative density units.

### In Vivo Experiments

The European Union Directive 2010/63/EU, established on September 22, 2010, outlines the criteria for the use of animals in scientific research. According to this directive, zebrafish (*D. rerio*) embryos and their early developmental stages are classified similarly to in vitro cell cultures. As a result, they are exempt from the regulations governing animal experiments. Since our in vivo research involved only zebrafish larvae younger than 120 h postfertilization (hpf), ethical approval was not necessary. The experimental conditions were as follows: temperature of 28.5 °C with a light/dark cycle of 14/10 h. Feeding protocols followed the guidelines of the Research Animals Department of the Royal Society for the Prevention of Cruelty to Animals (RSPCA). MCF-7 (HTB-22) and T-47D (HTB-133) ER-positive breast cancer cell lines (ATCC, Manassas, VA, USA) were seeded onto 6-well cell culture plates at a density of 5 × 10^5^ cells/ml. After 24 h, the culture medium was removed, and cells were treated with fisetin (F) and fisetin derivatives (FCPh_2_ and mF3) at concentrations of 1 and 5 μM for 48 h. Cells were washed with phosphate-buffered saline (PBS without Ca^2+^ and Mg^2+^) and then harvested. After detaching, cells were centrifuged (500*g*), washed with PBS, and labeled with Vybrant DiD (Thermo Fisher Scientific, Waltham, MA, USA) according to the manufacturer’s instructions. The stained cells were then resuspended in DMEM at a final concentration of 1 × 10^7^ cells/ml. Zebrafish larvae were manually dechorionated and subjected to injection at 2 days postfertilization (dpf). Approximately 300 labeled cells were introduced into the larval yolk cavity using an electronically controlled air-pressure microinjector (Narishige IM-300 Microinjector). Following injection, the zebrafish larvae with transplanted cells were placed in individual wells of a 24-well plate. The zebrafish with the xenografts were cultured in an E3 buffer at 32 °C until the end of the experiments (120 hpf). Prior to imaging, zebrafish larvae were subjected to anesthesia using 0.04 mg/mL ethyl 3-aminobenzoate methanesulfonate tricaine. Imaging of xenografts was performed at 2 and 5 dpf using an EVOS M5000 Imaging System equipped with a Cy5 filter (excitation: 628 nm; emission: 692 nm). Fold change (FC) images of the same fish on days 2 and 5 were used to compare the size of the tumor. ImageJ software was used to analyze the size of the tumor. Total RNA from zebrafish xenografts was extracted using the RNeasy Plus Kit (Qiagen, Hilden, Germany) following the manufacturer’s protocol. Subsequently, isolated RNA was used for cDNA synthesis using High-Capacity cDNA Reverse Transcription Kit with the addition of an RNase Inhibitor (Thermo Fisher Scientific, Waltham, MA, USA). The following primers were used: *CDKN1A* (p21) (forward 5′-AGGTGGACCTGGAGACTCTCAG-3′, reverse 5′-TCCTCTTGGAGAAGATCAGCCG-3′) and *BAX* (forward 5′-TCAGGATGCGTCCACCAAGAAG-3′, reverse 5′-TGTGTCCACGGCGGCAATCATC-3′). The human *GAPDH* gene (forward 5′-CTCTGCTCCTCCTGTTCGAC-3′, reverse 5′-GCCCAATACGACCAAATCC-3′) was used as a housekeeping gene. Quantitative real-time PCR (qPCR) expression analysis was performed using a PowerUp SYBR Green Master Mix (Thermo Fisher Scientific, Waltham, MA, USA) and LightCycler 480 II instrument (Roche, Basel, Switzerland) (96 well plate format, in triplicates). Relative mRNA expression was calculated using delta CT subtraction and normalized.

### Statistical Analysis

The results are calculated as the arithmetic mean ± standard deviation from three independent biological samples. Furthermore, if needed, box and whisker plots with median, lowest, and highest values were also applied. Treated and nontreated cells were compared using one-way analysis of variance (ANOVA) and Dunnett’s multiple comparison test (GraphPad Prism 8). Differences between fisetin and mito-fisetin-treated samples were also analyzed using one-way ANOVA and Tukey’s multiple comparison test. Furthermore, differences between the two means (control versus treated sample) were assessed using Student’s *t*-test (GraphPad Prism 8). *P* values of less than 0.05 were assumed as statistically significant.

## Data Availability

The data presented in this study are available in the Supporting Information.

## Supporting Information Available

General Information on materials and methods; synthesis of mF3 and mF7 compounds: mF3–synthetic pathway and mF7–synthetic pathway; overall synthetic pathway for mF3; synthesis of FCPh_2_; derivative of fisetin with 4-chlorobutoxy residue at position 3; acetylation at position 7; exchange of terminal halogen atom in chlorobutyloxy residue; exchange of terminal iodine atom into triphenylphosphonium iodide; overall synthetic pathway for mF7; acetylation of hydroxy groups in position 3 and 7 of fisetin; derivative of fisetin with 4-chlorobutoxy residue at position 7; exchange of terminal halogen atom in chlorobutyl residue; exchange of terminal iodine atom into triphenylphosphonium iodide; ^1^H and ^13^C NMR spectra of compounds; normal phase isocratic HPLC profiles for mF3 and mF7; changes in the metabolic activity of ER-positive breast cancer cells upon stimulation with tamoxifen; fisetin derivative-mediated changes in mitochondrial parameters in proliferating noncancerous MCF10F cells and HCC1428 breast cancer cells; mF3-mediated apoptosis in BJ human fibroblasts; effect of mF3 on the levels of key markers of necroptotic cell death in HCC1428 breast cancer cells; and Western blot-based analysis of the levels of Bcl-2, Bcl-rambo, AKT, and phospho-AKT in mF3-treated proliferating normal MCF10F cells and HCC1428 breast cancer cells (PDF)
Molecular formula strings for mF3 and mF7 (CSV)

## Abbreviations

AMPK: AMP-activated protein kinase
BECN1: beclin 1
BNIP3: Bcl-2/adenovirus E1B 19 kDa interacting protein 3
DMEM: Dulbecco’s modified Eagle medium
ER: estrogen receptor
F: fisetin
FBS: fetal bovine serum
FCCP: carbonyl cyanide 4-(trifluoromethoxy) phenylhydrazone
FCPh_2_: 2-(2,2-diphenylbenzo[*d*][1,3]dioxol-5-yl)-3,7-dihydroxy-4*H*-chromen-4-one
FUNDC1: FUN14 domain-containing protein 1
HSP: heat shock protein
IL-8: interleukin 8
LC3B: microtubule-associated protein 1 light chain 3 beta
LIR: LC3-interacting region
mF3: (4-((7-acetoxy-2-(2,2-diphenylbenzo[*d*][1,3]dioxol-5-yl)-4-oxo-4*H*-chromen-3-yl)oxy)butyl) triphenylphosphonium iodide
mF7: (4-((3,5-diacetoxy-2-(2,2-diphenylbenzo[*d*][1,3]dioxol-5-yl)-4-oxo-4*H*-chromen-7-yl)oxy)butyl) triphenylphosphonium iodide
MMP: mitochondrial membrane potential
NIX: NIP-3-like protein X
OCR: oxygen consumption rate
OMM: outer mitochondrial membrane
OXPHOS: oxidative phosphorylation
PBS: phosphate-buffered saline
PCR: polymerase chain reaction
PINK1: PTEN-induced putative kinase 1
RFU: relative fluorescent units
RPMI: Roswell Park Memorial Institute
SERM: selective estrogen receptor modulator
SOD: superoxide dismutase
TAM: tamoxifen
TNBC: triple negative breast cancer
TPP^+^ groups: triphenyl phosphonium cationic groups
ULK1: unc-51 like autophagy activating kinase 1
VPS34: phosphoinositide-3-kinase class 3
