# Supplementary material for: New Mitochondria-Targeted Fisetin Derivative Compromises Mitophagy and Limits Survival of Drug-Induced Senescent Breast Cancer Cells
Source: J Med Chem. 2024 Sep 25;67(19):17676–89. doi: 10.1021/acs.jmedchem.4c01664 (PMC11472315; doi:10.1021/acs.jmedchem.4c01664)
Supplement: Supplementary file 1 — jm4c01664_si_001.pdf [file jm4c01664_si_001.pdf]

Supporting Information

A new mitochondria-targeted fisetin derivative compromises mitophagy and limits survival of drug-induced senescent breast cancer cells

Iwona Rzeszutek[a#], Martyna Cybularczyk-Cecotka[b#], Anna Deręgowska[a], Paulina Stec[a], Maciej Wnuk[a], Olga Kołodziej[a], Joanna Kałafut[c], Anna Wawruszak[c], Wojciech Witkowski[b], Grzegorz Litwinienko[b*], Anna Lewińska[a*]

[a]*Institute of Biotechnology, College of Natural Sciences, University of Rzeszow, Pigonia 1, 35-310 Rzeszow, Poland*

[b]*Faculty of Chemistry, University of Warsaw, Pasteura 1, 02-093 Warsaw, Poland*

[c]*Department of Biochemistry and Molecular Biology, Medical University of Lublin, Chodźki 1, 20-093 Lublin, Poland*

[#]Contributed equally

*Correspondence: Grzegorz Litwinienko (litwin@chem.uw.edu.pl), Anna Lewińska (alewinska@ur.edu.pl).

Table of Contents

	Page
1. General Information on materials and methods.	S-3
2. Synthesis of mF3 and mF7 compounds.	S-3
A) mF3 – synthetic pathway.	S-3
**Scheme S1.** Overall synthetic pathway for mF3.	S-3
**Scheme S2.** Synthesis of $FCPh_2$.	S-4
**Scheme S3.** Derivative of fisetin with 4-chlorobutoxy residue at position 3.	S-4
**Scheme S4.** Acetylation at position 7.	S-5
**Scheme S5.** Exchange of terminal halogen atom in chlorobutyloxy residue.	S-6
**Scheme S6.** Exchange of terminal iodine atom into triphenylphosphonium iodide.	S-7
B) mF7 – synthetic pathway.	S-8
**Scheme S7.** Overall synthetic pathway for mF7.	S-8
**Scheme S8.** Acetylation of hydroxy groups in position 3 and 7 of fisetin.	S-9
**Scheme S9.** Derivative of fisetin with 4-chlorobutoxy residue at position 7.	S-10
**Scheme S10.** Exchange of terminal halogen atom in chlorobutyl residue.	S-11
**Scheme S11.** Exchange of terminal iodine atom into triphenylphosphonium iodide.	S-12
**Figures S1-S18**. ^{1}H and ^{13}C NMR spectra of compounds.	S-13
**Figure S19.** Normal phase isocratic HPLC profiles for mF3 and mF7.	S-31
**Figure S20.** Changes in the metabolic activity of ER-positive breast cancer cells (HCC1500, CAMA-1, HCC1428, and ZR-75-30) upon stimulation with tamoxifen (TAM).	S-32
**Figure S21.** Fisetin derivative-mediated changes in mitochondrial parameters in proliferating non-cancerous MCF10F cells and HCC1428 breast cancer cells.	S-33
**Figure S22.** Mito-fisetin (mF3)-mediated apoptosis in BJ human fibroblasts.	S-35
**Figure S23.** The effect of mito-fisetin (mF3) on the levels of key markers of necroptotic cell death in HCC1428 breast cancer cells.	S-36
**Figure S24.** Western blot-based analysis of the levels of Bcl-2, Bcl-rambo, AKT, and phospho-AKT in mF3-treated proliferating normal MCF10F cells and HCC1428 breast cancer cells.	S-37

1. General Information on materials and methods

All reagents and solvents were purchased from commercial suppliers and used without further purification. Thin layer chromatography (TLC) was performed using Merck Silica Gel F254, 0.20 mm thickness and the visualization was accomplished by irradiation at 254 nm. All aqueous solutions were prepared using distilled water. Saturated brine refers to an aqueous saturated sodium chloride solution. All products were purified by column chromatography using silica gel 60 M (40-63 μm, 230-440 mesh). NMR spectra were recorded at room temperature using Bruker 300 MHz spectrometer. Chemical shifts are reported relatively in δ-scale as parts per million (ppm) referenced to the residual solvent peak. Coupling constants *J* are given in Hertz (Hz) and the following abbreviations were used for indicating signal multiplicity: ^{1}H NMR: s = singlet, d = doublet, t = triplet, q = quartet, hept = heptet, m = multiplet and the respective combinations.

2. Synthesis of mF3 and mF7 compounds

A) mF3 – synthetic pathway

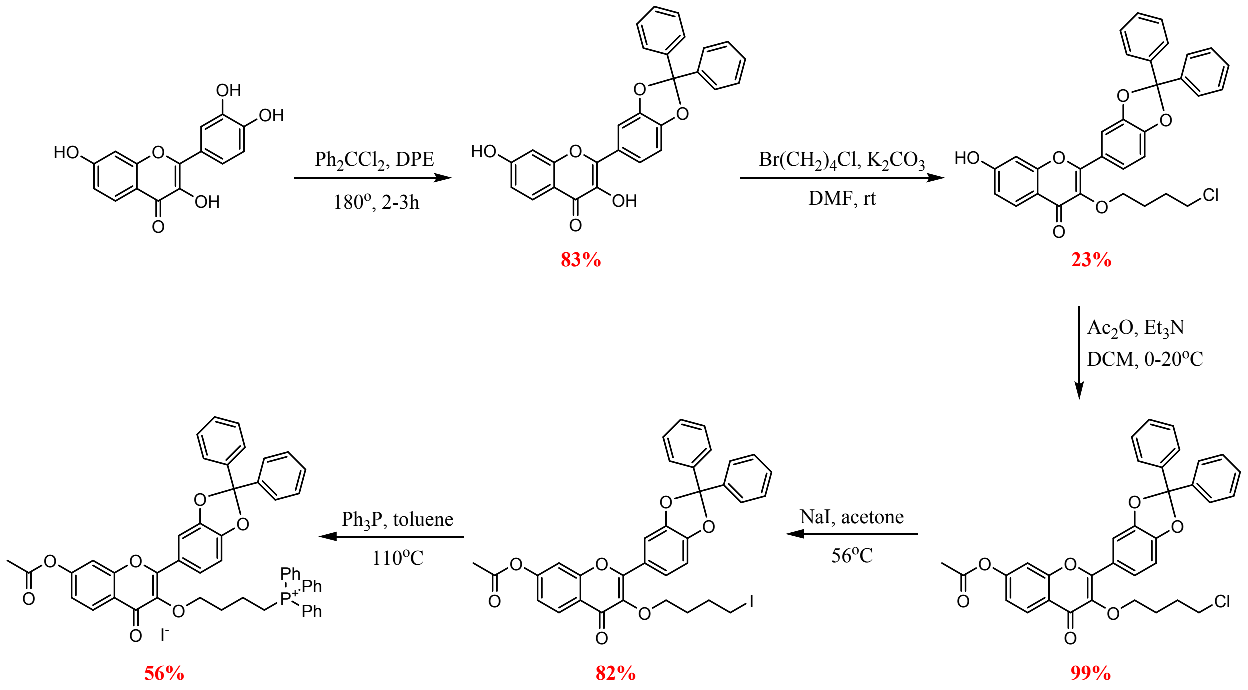

Scheme S1. Overall synthetic pathway for mF3.

2-(2,2-diphenylbenzo[*d*][1,3]dioxol-5-yl)-3,7-dihydroxy-4H-chromen-4-one ($FCPh_2$)

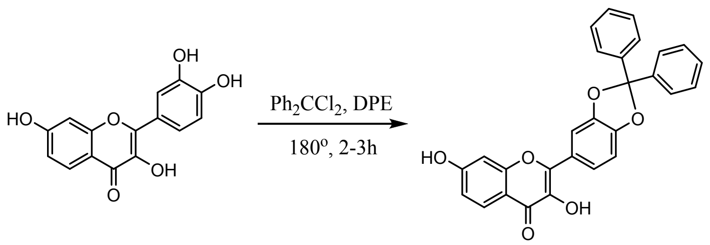

Scheme S2. Synthesis of $FCPh_2$.

Fisetin (500 mg, 1.75 mmol) and 1,1-dichlorodiphenylmethane (621 mg, 2.62 mmol) were dissolved in diphenyl ether (20 ml) and the reaction mixture was heated to 182 °C with stirring. After 5 h, the mixture was cooled to room temperature, petroleum ether (50 ml) was added. The dark yellow crude product was obtained by filtration and purified by silica gel column chromatography (20-30% ethyl acetate in petroleum ether as eluent) to give a product as a light-yellow solid with isolated yield: 83% (653 mg, 1.45 mmol). ^{1}H NMR (300 MHz, DMSO-d_6) δ 10.79 (s, 1H), 9.33 (s, 1H), 7.93 (d, J = 8.7 Hz, 1H), 7.89 – 7.77 (m, 2H), 7.66 – 7.52 (m, 4H), 7.52 – 7.38 (m, 7H), 7.21 (d, J = 8.4 Hz, 1H), 7.00 – 6.85 (m, 2H). ^{13}C NMR (75 MHz, DMSO) δ 172.1, 162.5, 156.4, 147.3, 146.7, 144.0, 139.5, 137.8, 129.5, 128.7, 126.5, 125.8, 125.8, 122.7, 117.0, 114.9, 114.2, 108.9, 107.7, 102.1.

3-(4-chlorobutoxy)-2-(2,2-diphenylbenzo[*d*][1,3]dioxol-5-yl)-7-hydroxy-4H-chromen-4-one

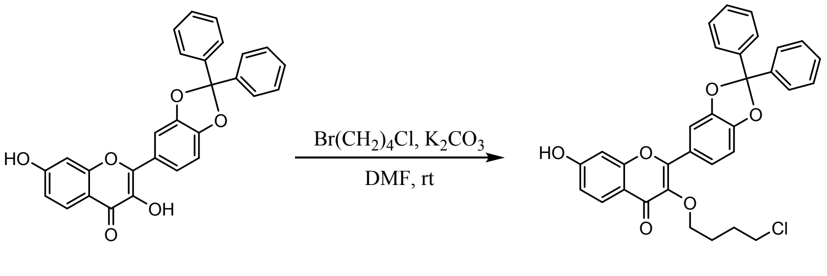

Scheme S3. Derivative of fisetin with 4-chlorobutoxy residue at position 3.

$FCPh_2$ (653 mg, 1.45 mmol) and K_2CO_3 (181 mg, 1.31 mmol) were dissolved in DMF (10 ml) and 1-bromo-4-chlorobutane (225 mg, 1.31 mmol) was added. The reaction mixture was stirred overnight at RT. After confirming by TLC that all substrate had reacted, the reaction mixture was diluted with ethyl acetate (100 ml), transfer into the separating funnel and washed 3 times with 50 ml of 1N HCl. Organic layer was dried with anhydrous $MgSO_4$. The crude product was purified by column chromatography with elucidation by petroleum ether/DCM/acetone (8:1:1) mixture to give 3-(4-chlorobutoxy)-2-(2,2-diphenylbenzo[*d*][1,3]dioxol-5-yl)-7-hydroxy-4H-chromen-4-one as light-yellow oil with isolated yield 23% (179 mg, 0.33 mmol). ^{1}H NMR (300 MHz, $CDCl_3$) δ 7.99 – 7.81 (m, 2H), 7.73 (d, *J* = 1.7 Hz, 1H), 7.69 – 7.52 (m, 5H), 7.51 – 7.33 (m, 7H), 7.00 (d, *J* = 8.2 Hz, 1H), 6.69 (dd, *J* = 8.7, 2.4 Hz, 1H), 6.56 (d, *J* = 2.4 Hz, 1H), 4.16 (t, *J* = 6.1 Hz, 2H), 3.34 (t, *J* = 6.2 Hz, 2H), 1.75 – 1.58 (m, 4H). ^{13}C NMR (75 MHz, $CDCl_3$) δ 165.6, 164.8, 161.6, 152.3, 152.1, 147.7, 139.6, 133.9, 129.6, 128.5, 126.8, 126.3, 123.1, 118.5, 115.1, 113.8, 111.2, 110.4, 108.6, 64.3, 44.4, 29.8, 29.1, 26.0.

3-(4-chlorobutoxy)-2-(2,2-diphenylbenzo[d][1,3]dioxol-5-yl)-4-oxo-4H-chromen-7-yl acetate

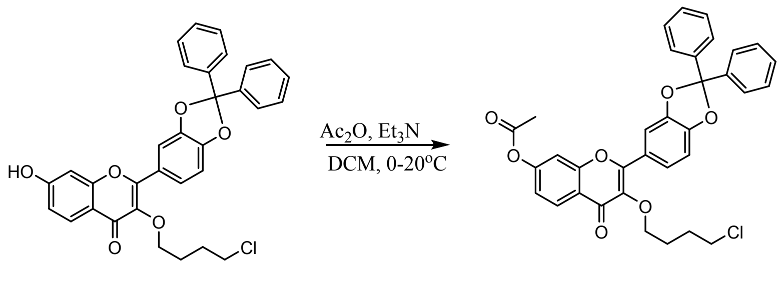

Scheme S4. Acetylation at position 7.

3-(4-chlorobutoxy)-2-(2,2-diphenylbenzo[*d*][1,3]dioxol-5-yl)-4-oxo-4H-chromen-7-yl acetate (179 mg, 0.33 mmol) was dissolved in 20 ml DCM and Et_3N (55 μl, 0.40 mmol) was added. The solution was cooled to 0°C and acetic anhydride (33 μl, 0.33 mmol) was added dropwise.

The reaction mixture was allowed to warm up to RT and it was stirred until starting material disappeared completely (monitored by TLC, with petroleum ether/acetone 8:2 as eluent). After finishing, the reaction mixture was diluted by DCM and extracted 3 times with 1N HCl. Organic phase was dried over anhydrous $MgSO_4$. After solvent evaporation, the crude product in the form of bright yellow oil turned out to be sufficiently pure to be used in next synthesis stage without further purification. Isolated yield: 99% (190 mg, 0.33 mmol). ^{1}H NMR (300 MHz, $CDCl_3$) δ 8.09 (d, J = 8.6 Hz, 1H), 7.85 (dd, J = 8.2, 1.7 Hz, 1H), 7.70 (d, J = 1.7 Hz, 1H), 7.65 – 7.54 (m, 5H), 7.46 – 7.35 (m, 7H), 7.13 (dd, J = 8.7, 2.3 Hz, 1H), 7.04 (d, J = 2.2 Hz, 1H), 6.99 (d, J = 8.3 Hz, 1H), 4.19 (t, J = 6.1 Hz, 2H), 3.33 (t, J = 6.2 Hz, 2H), 2.31 (s, 3H), 1.78 – 1.57 (m, 4H).^{13}C NMR (75 MHz, $CDCl_3$) δ 168.5, 164.4, 164.2, 154.6, 152.0, 151.7, 147.7, 139.6, 133.0, 129.6, 128.5, 126.6, 126.4, 123.1, 121.3, 119.3, 118.5, 117.7, 110.4, 108.6, 64.6, 44.4, 29.1, 26.0, 21.3.

2-(2,2-diphenylbenzo[*d*][1,3]dioxol-5-yl)-3-(4-iodobutoxy)-4-oxo-4H-chromen-7-yl acetate

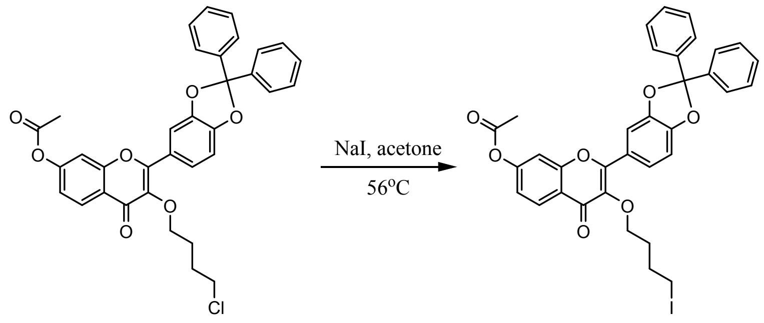

Scheme S5. Exchange of terminal halogen atom in chlorobutyloxy residue.

3-(4-chlorobutoxy)-2-(2,2-diphenylbenzo[d][1,3]dioxol-5-yl)-4-oxo-4H-chromen-7-yl acetate (190 mg, 0.33 mmol) and NaI (989 mg, 6.6 mmol) were dissolved in acetone (5 ml) and heated at reflux overnight. Next day, small amount of solution was taken, evaporated under reduced pressure and measured by ^{1}H NMR to calculate the reaction conversion (Rf of substrate and product are the same). If there were no substrate traces on ^{1}H NMR spectrum,

the reaction mixture was cooled, diluted with 100 ml of ethyl acetate, filtered through paper filter directly into the separating funnel and washed 3 times with 50 ml of water. Organic layer was dried with anhydrous $MgSO_4$. The crude product was purified by column chromatography with elucidation by petroleum ether/acetone 8:2 mixture to give 2-(2,2-diphenylbenzo[*d*][1,3]dioxol-5-yl)-3-(4-iodobutoxy)-4-oxo-4H-chromen-7-yl acetate as light yellow solid with isolated yield 82% (183 g, 0.27 mmol). ^{1}H NMR (300 MHz, $CDCl_3$) δ 8.14 – 8.06 (m, 1H), 7.85 (dd, *J* = 8.2, 1.7 Hz, 1H), 7.74 – 7.69 (m, 1H), 7.66 – 7.55 (m, 4H), 7.46 – 7.37 (m, 6H), 7.14 (dd, *J* = 8.7, 2.3 Hz, 1H), 7.07 – 6.96 (m, 2H), 4.17 (t, *J* = 6.3 Hz, 2H), 2.96 (t, *J* = 6.8 Hz, 2H), 2.31 (s, 3H), 1.81 – 1.66 (m, 2H), 1.64 – 1.51 (m, 2H). ^{13}C NMR (75 MHz, $CDCl_3$) δ 168.5, 164.3, 164.2, 154.5, 152.0, 151.7, 147.6, 139.6, 133.0, 129.6, 128.5, 126.6, 126.3, 123.1, 121.3, 119.3, 118.5, 117.6, 110.3, 108.6, 64.2, 44.4, 29.9, 29.5, 21.2, 5.8.

(4-((7-acetoxy-2-(2,2-diphenylbenzo[*d*][1,3]dioxol-5-yl)-4-oxo-4H-chromen-3-yl)oxy)butyl)triphenylphosphonium iodide

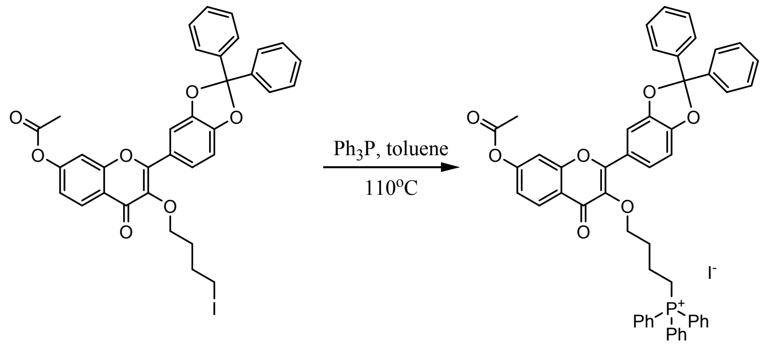

Scheme S6. Exchange of terminal iodine atom into triphenylphosphonium iodide.

2-(2,2-diphenylbenzo[*d*][1,3]dioxol-5-yl)-3-(4-iodobutoxy)-4-oxo-4H-chromen-7-yl acetate (183 mg, 0.27 mmol) and Ph_3P (1.09 g, 1.35 mmol) were dissolved in toluene (20 ml) and heated at reflux for 1-3 days with monitoring the reaction course by TLC. When all the substrate had reacted, the reaction mixture was cooled and the solvent was evaporated under reduced pressure. The crude product was purified by column chromatography with

elucidation by DCM/methanol (96:4) mixture to give yellow solid of (4-((7-acetoxy-2-(2,2-diphenylbenzo[*d*][1,3]dioxol-5-yl)-4-oxo-4H-chromen-3-yl)oxy)butyl)triphenylphosphonium iodide (abbreviated in the manuscript as mF3) with isolated yield 56% (142 g, 0.15 mmol). ^{1}H NMR (300 MHz, $CDCl_3$) δ 7.90 (dd, *J* = 8.6, 0.3 Hz, 1H), 7.81 – 7.67 (m, 10H), 7.67 – 7.51 (m, 11H), 7.49 (dd, *J* = 1.8, 0.4 Hz, 1H), 7.45 – 7.34 (m, 6H), 7.07 (dd, *J* = 8.6, 2.3 Hz, 1H), 6.99 (d, *J* = 2.2 Hz, 1H), 6.92 (dd, *J* = 8.2, 0.3 Hz, 1H), 4.27 (t, *J* = 5.7 Hz, 2H), 3.80 – 3.61 (m, 2H), 2.33 (s, 3H), 2.02 (p, *J* = 6.5 Hz, 2H), 1.65 (q, *J* = 7.8 Hz, 2H). ^{13}C NMR (75 MHz, $CDCl_3$) δ 168.6, 164.3, 163.8, 154.5, 152.0, 151.7, 147.6, 139.5, 135.2, 135.1, 133.8, 133.6, 132.7, 130.6, 130.5, 130.5, 129.6, 128.5, 126.4, 126.3, 122.9, 121.0, 119.4, 118.5, 118.5, 117.6, 117.3, 110.1, 108.6, 63.6, 29.7, 28.9, 28.6, 23.1, 21.9, 21.2, 19.0.

B) mF7 – synthetic pathway

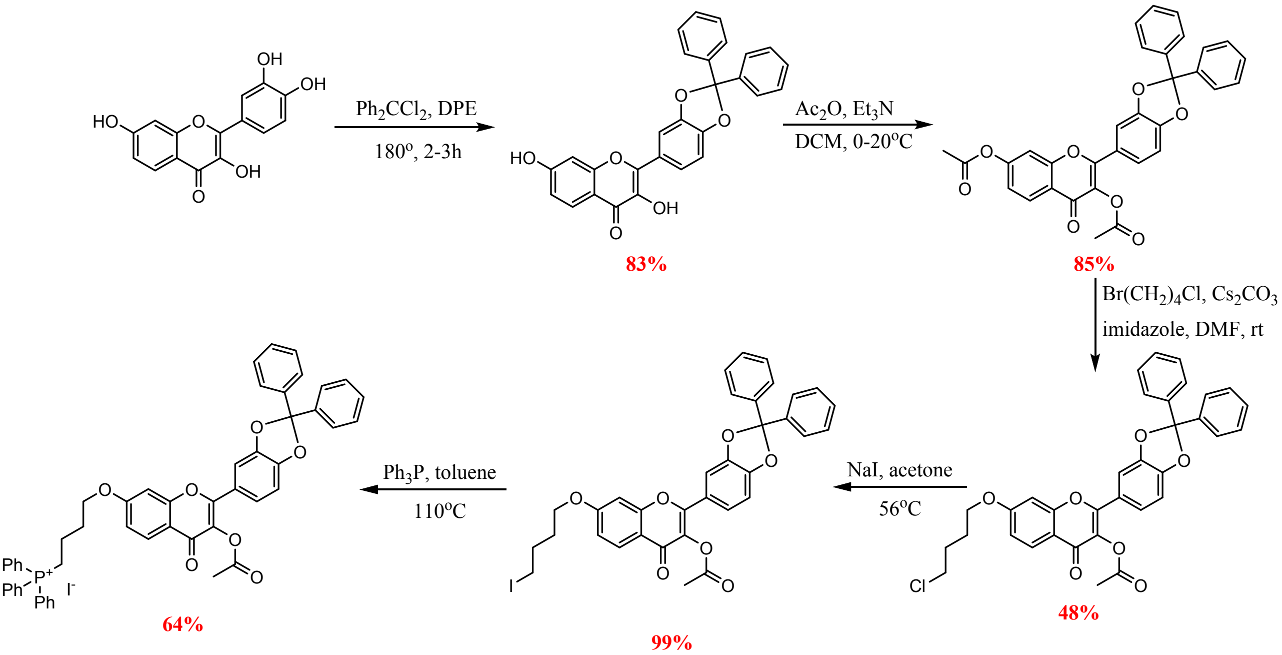

Scheme S7. Overall synthetic pathway for mF7.

2-(2,2-diphenylbenzo[*d*][1,3]dioxol-5-yl)-4-oxo-4H-chromene-3,7-diyl diacetate

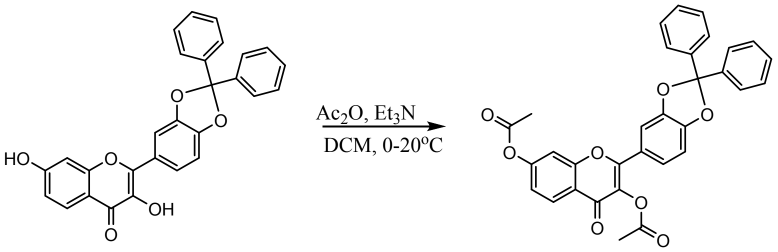

Scheme S8. Acetylation of hydroxy groups in position 3 and 7 of fisetin.

$FCPh_2$ (150 mg, 0.30 mmol) was dissolved in 10 ml of DCM and Et_3N (0.42 ml, 3.1 mmol) was added. The solution was cooled to 0°C and acetic anhydride (0.12 ml, 1.22 mmol) was added dropwise. The reaction mixture was allowed to warm up to RT and it was stirred until starting material disappeared completely (monitored by TLC, with petroleum ether/acetone 8:2 as eluent). After finishing, the reaction mixture was diluted by DCM and extracted 3 times with 1N HCl. Organic phase was dried over anhydrous $MgSO_4$. After solvent evaporation, the crude product was purified by column chromatography with elucidation by petroleum ether/ethyl acetate 7:3 mixture to give pure 2-(2,2-diphenylbenzo[*d*][1,3]dioxol-5-yl)-4-oxo-4H-chromene-3,7-diyl diacetate as white solid with isolated yield 85% (138 mg, 0.26 mmol). ^1H NMR (300 MHz, $CDCl_3$) δ 8.25 (dd, *J* = 8.8, 0.4 Hz, 1H), 7.66 – 7.54 (m, 4H), 7.51 – 7.34 (m, 9H), 7.16 (dd, *J* = 8.7, 2.2 Hz, 1H), 7.00 (dd, *J* = 8.2, 0.4 Hz, 1H), 2.36 (s, 3H), 2.36 (s, 3H). ^{13}C NMR (75 MHz, $CDCl_3$) δ 171.6, 168.6, 168.1, 156.3, 156.0, 154.8, 149.9, 147.8, 139.7, 133.4, 129.6, 128.5, 127.6, 126.4, 123.8, 123.5, 121.4, 119.6, 118.3, 111.0, 108.9, 108.5, 29.8, 21.3, 20.8.

7-(4-chlorobutoxy)-2-(2,2-diphenylbenzo[*d*][1,3]dioxol-5-yl)-4-oxo-4H-chromen-3-yl acetate

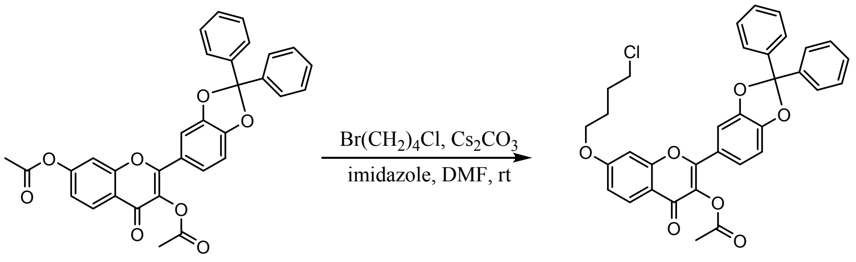

Scheme S9. Derivative of fisetin with 4-chlorobutoxy residue at position 7.

2-(2,2-diphenylbenzo[d][1,3]dioxol-5-yl)-4-oxo-4H-chromene-3,7-diyl diacetate (138 mg, 0.26 mmol), Cs_2CO_3 (127 mg, 0.39 mmol) and 1-bromo-4-chlorobutane (134 mg, 0.78 mmol) were dissolved in DMF (5 ml) and catalytic amount of imidazole was added. The reaction mixture was stirred 2 days under nitrogen atmosphere at RT. After confirming by TLC that all substrate had reacted, the reaction mixture was diluted with ethyl acetate (100 ml), transfer into the separating funnel and washed 3 times with 50 ml of 1N HCl. Organic layer was dried with anhydrous $MgSO_4$. The solvent was evaporated and crude product was purified by column chromatography with elucidation by petroleum ether/DCM/ethyl acetate 74/13/13 mixture to give 7-(4-chlorobutoxy)-2-(2,2-diphenylbenzo[d][1,3]dioxol-5-yl)-4-oxo-4H-chromen-3-yl acetate as white solid. Isolated yield: 48% (73 mg, 0.12 mmol). ^{1}H NMR (300 MHz, $CDCl_3$) δ 8.12 (d, J = 8.9 Hz, 1H), 7.65 – 7.55 (m, 4H), 7.47 – 7.35 (m, 8H), 6.98 (ddd, J = 9.0, 7.2, 1.4 Hz, 2H), 6.88 (d, J = 2.3 Hz, 1H), 4.10 (td, J = 5.5, 4.8, 2.0 Hz, 2H), 3.72 – 3.58 (m, 2H), 2.35 (s, 3H), 2.09 – 1.94 (m, 4H). ^{13}C NMR (75 MHz, $CDCl_3$) δ 171.6, 168.3, 163.7, 157.4, 155.5, 149.7, 147.8, 139.8, 133.3, 129.6, 128.5, 127.6, 126.4, 123.9, 123.6, 118.2, 117.5, 115.1, 108.8, 108.5, 100.7, 67.9, 44.7, 29.3, 26.5, 20.9.

2-(2,2-diphenylbenzo[*d*][1,3]dioxol-5-yl)-7-(4-iodobutoxy)-4-oxo-4H-chromen-3-yl acetate

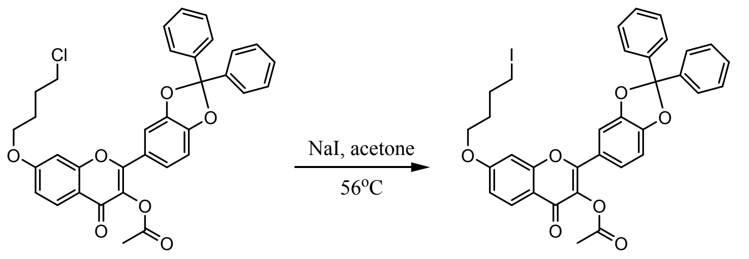

Scheme S10. Exchange of terminal halogen atom in chlorobutyl residue.

7-(4-chlorobutoxy)-2-(2,2-diphenylbenzo[*d*][1,3]dioxol-5-yl)-4-oxo-4H-chromen-3-yl acetate (73 mg, 0.12 mmol) and NaI (360 mg, 2.40 mmol) were dissolved in acetone (3 ml) and heated at reflux overnight. Next day, small amount of solution was taken, evaporated under reduced pressure and measured by ^{1}H NMR to calculate the reaction conversion (Rf of substrate and product are the same). If there was no substrate traces on ^{1}H NMR spectrum, the reaction mixture was cooled, diluted with 100 ml of ethyl acetate, filtered through paper filter directly into the separating funnel and washed 3 times with 50 ml of water. Organic layer was dried with anhydrous $MgSO_4$. The crude product was purified by column chromatography with elucidation by petroleum ether/acetone 8:2 mixture to give 2-(2,2-diphenylbenzo[*d*][1,3]dioxol-5-yl)-7-(4-iodobutoxy)-4-oxo-4H-chromen-3-yl acetate as white solid with isolated yield 99% (83 mg, 0.12 mmol). ^{1}H NMR (300 MHz, $CDCl_3$) δ 8.12 (d, J = 8.9 Hz, 1H), 7.65 – 7.56 (m, 4H), 7.49 – 7.35 (m, 8H), 7.04 – 6.92 (m, 2H), 6.88 (d, J = 2.3 Hz, 1H), 4.06 (t, J = 5.8 Hz, 2H), 3.26 (t, J = 6.5 Hz, 2H), 2.35 (s, 3H), 2.09 – 1.89 (m, 5H). ^{13}C NMR (75 MHz, $CDCl_3$) δ 171.5, 168.2, 163.6, 157.3, 155.4, 149.6, 147.7, 139.7, 133.2, 129.5, 128.5, 127.4, 126.7, 123.8, 123.5, 118.2, 117.4, 115.0, 108.8, 108.4, 100.7, 67.5, 30.0, 29.9, 20.8, 6.1, 1.1.

(4-((3,5-diacetoxy-2-(2,2-diphenylbenzo[*d*][1,3]dioxol-5-yl)-4-oxo-4H-chromen-7-yl)oxy)butyl)triphenylphosphonium iodide

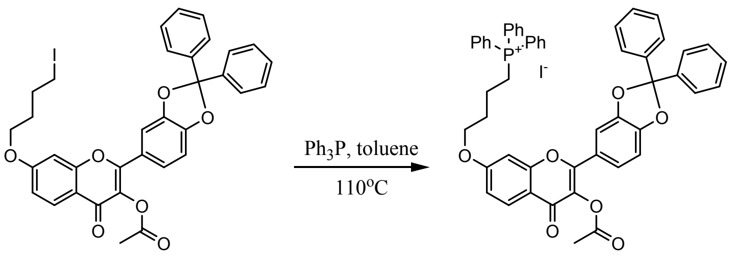

Scheme S11. Exchange of terminal iodine atom into triphenylphosphonium iodide.

2-(2,2-diphenylbenzo[*d*][1,3]dioxol-5-yl)-7-(4-iodobutoxy)-4-oxo-4H-chromen-3-yl acetate (83 mg, 0.12 mmol) and Ph_3P (157 mg, 0.60 mmol) were dissolved in toluene (10 ml) and heated at reflux for 24 h with monitoring the reaction course by TLC. When all the substrate had reacted, the reaction mixture was cooled and the solvent was evaporated under reduced pressure. The crude product was purified by column chromatography with elucidation by DCM/methanol 96:4 mixture to give yellow solid of (4-((3,5-diacetoxy-2-(2,2-diphenylbenzo[*d*][1,3]dioxol-5-yl)-4-oxo-4H-chromen-7-yl)oxy)butyl)triphenylphosphonium iodide (abbreviated in the manuscript as mF7) with isolated yield 64% (72 mg, 0.08 mmol). ^{1}H NMR (300 MHz, $CDCl_3$) δ 7.98 (d, J = 8.9 Hz, 1H), 7.86 – 7.70 (m, 9H), 7.70 – 7.60 (m, 6H), 7.60 – 7.51 (m, 4H), 7.44 (dd, J = 8.3, 1.8 Hz, 1H), 7.41 – 7.30 (m, 7H), 7.00 – 6.87 (m, 2H), 6.80 (dd, J = 8.9, 2.3 Hz, 1H), 4.17 (t, J = 5.6 Hz, 2H), 3.87 – 3.70 (m, 2H), 2.35 – 2.28 (m, 3H), 2.25 (td, J = 6.9, 3.8 Hz, 2H), 1.86 (t, J = 8.0 Hz, 2H). ^{13}C NMR (75 MHz, $CDCl_3$) δ 171.4, 168.1, 163.4, 157.2, 155.4, 149.6, 147.6, 139.6, 135.2, 135.2, 133.7, 133.6, 133.0, 130.6, 130.5, 129.4, 128.4, 127.1, 126.2, 123.6, 118.5, 118.1, 117.4, 117.2, 115.0, 108.7, 108.3, 100.8, 67.4, 31.0, 29.2, 29.0, 22.7, 22.1, 20.7, 19.2.

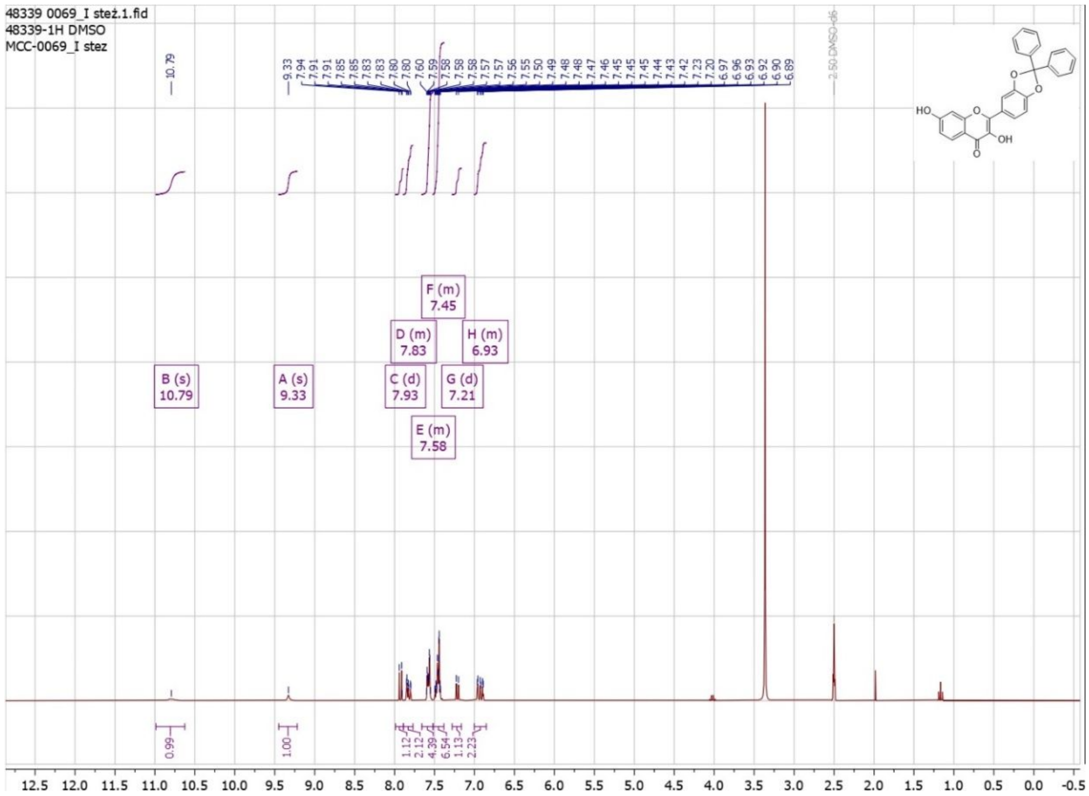

Figure S1. ^{1}H NMR (300 MHz, DMSO-d_6) of 2-(2,2-diphenylbenzo[*d*][1,3]dioxol-5-yl)-3,7-dihydroxy-4H-chromen-4-one ($FCPh_2$).

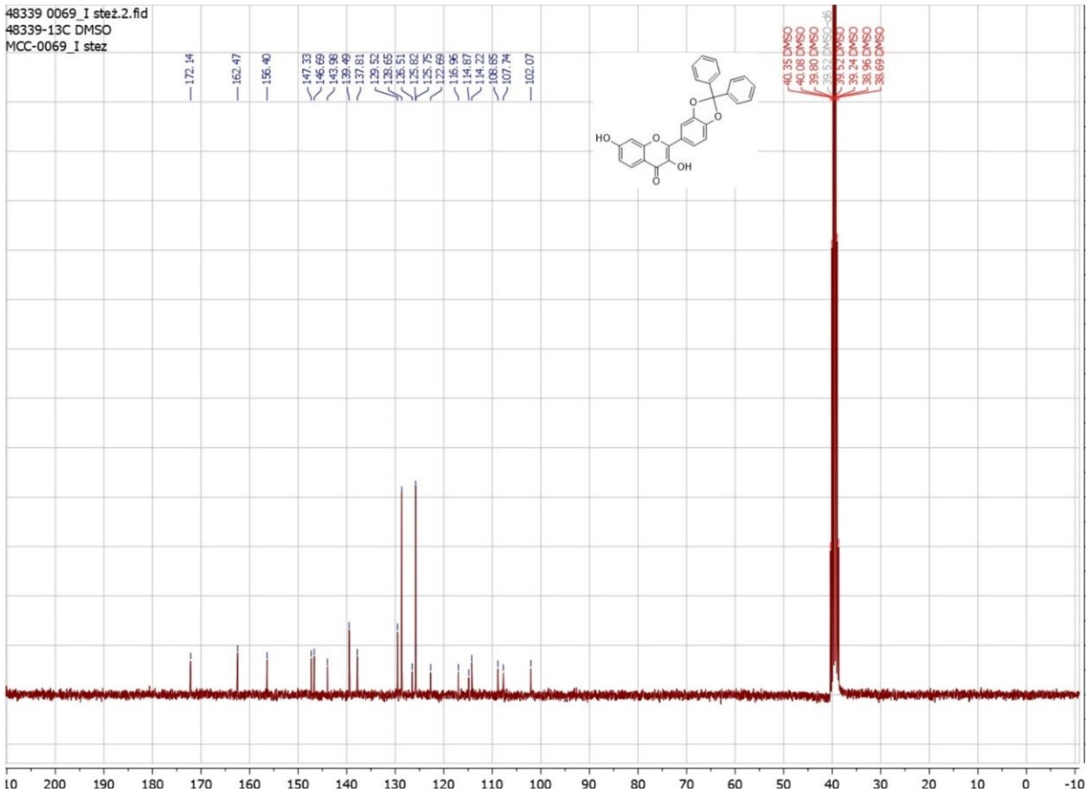

Figure S2. ^{13}C NMR (75 MHz, DMSO-d_6) of 2-(2,2-diphenylbenzo[*d*][1,3]dioxol-5-yl)-3,7-dihydroxy-4H-chromen-4-one.

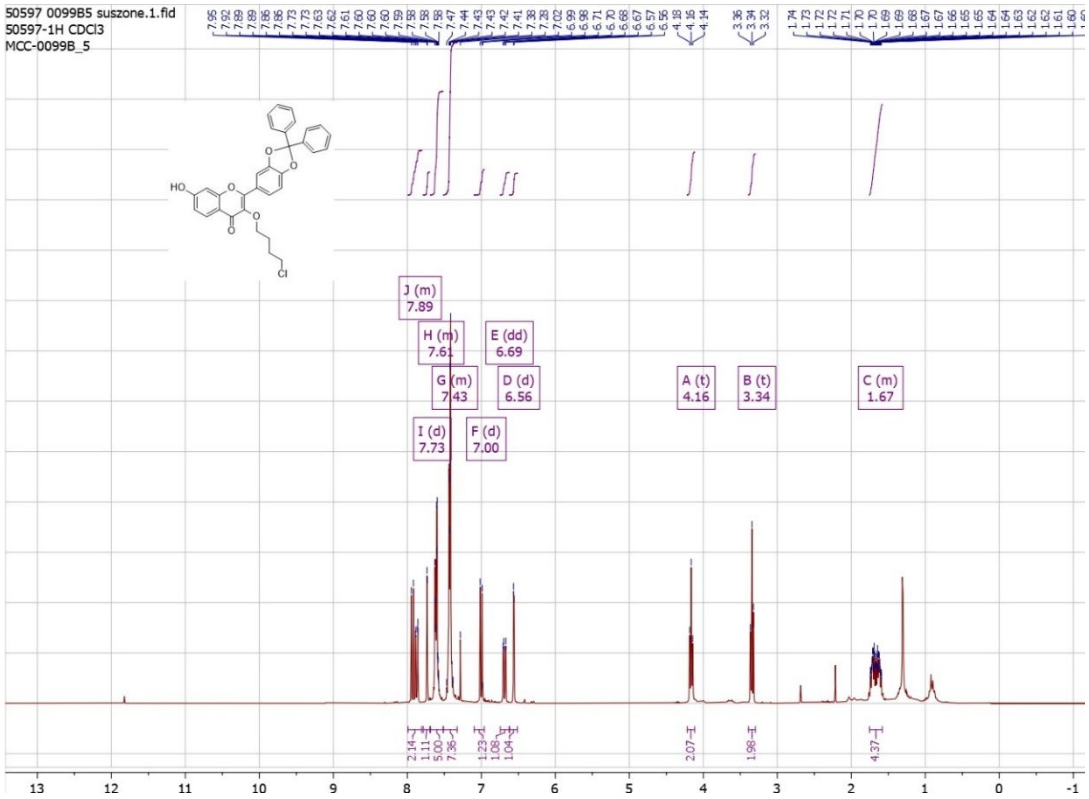

Figure S3. ^{1}H NMR (300 MHz, $CDCl_3$) of 3-(4-chlorobutoxy)-2-(2,2-diphenylbenzo[*d*][1,3]dioxol-5-yl)-7-hydroxy-4H-chromen-4-one.

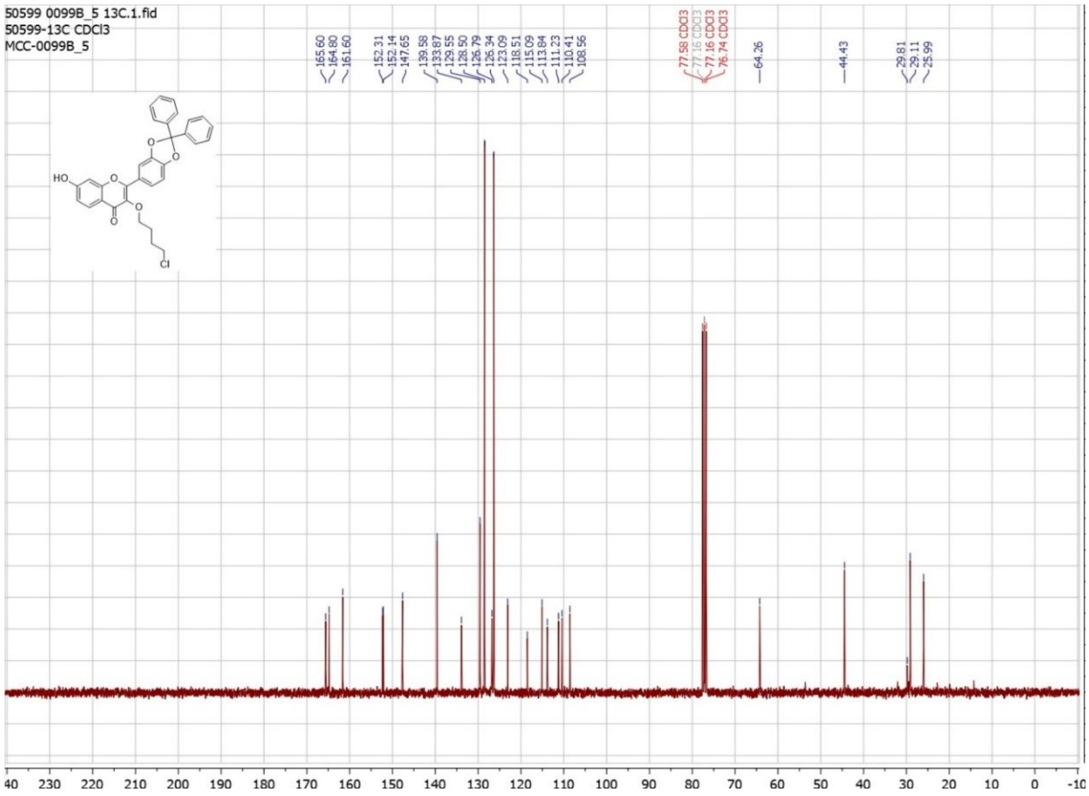

Figure S4. ^{13}C NMR (75 MHz, $CDCl_3$) of 3-(4-chlorobutoxy)-2-(2,2-diphenylbenzo[*d*][1,3]dioxol-5-yl)-7-hydroxy-4H-chromen-4-one.

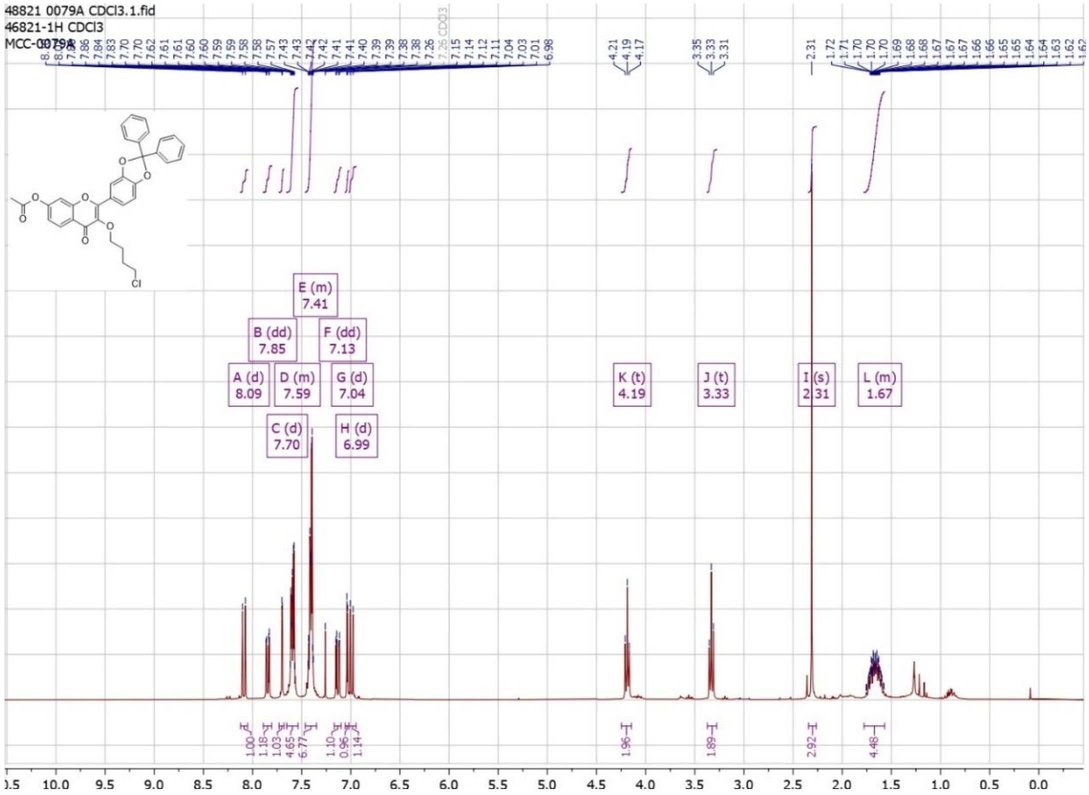

Figure S5. ^{1}H NMR (300 MHz, $CDCl_3$) of 3-(4-chlorobutoxy)-2-(2,2-diphenylbenzo[*d*][1,3]dioxol-5-yl)-4-oxo-4H-chromen-7-yl acetate.

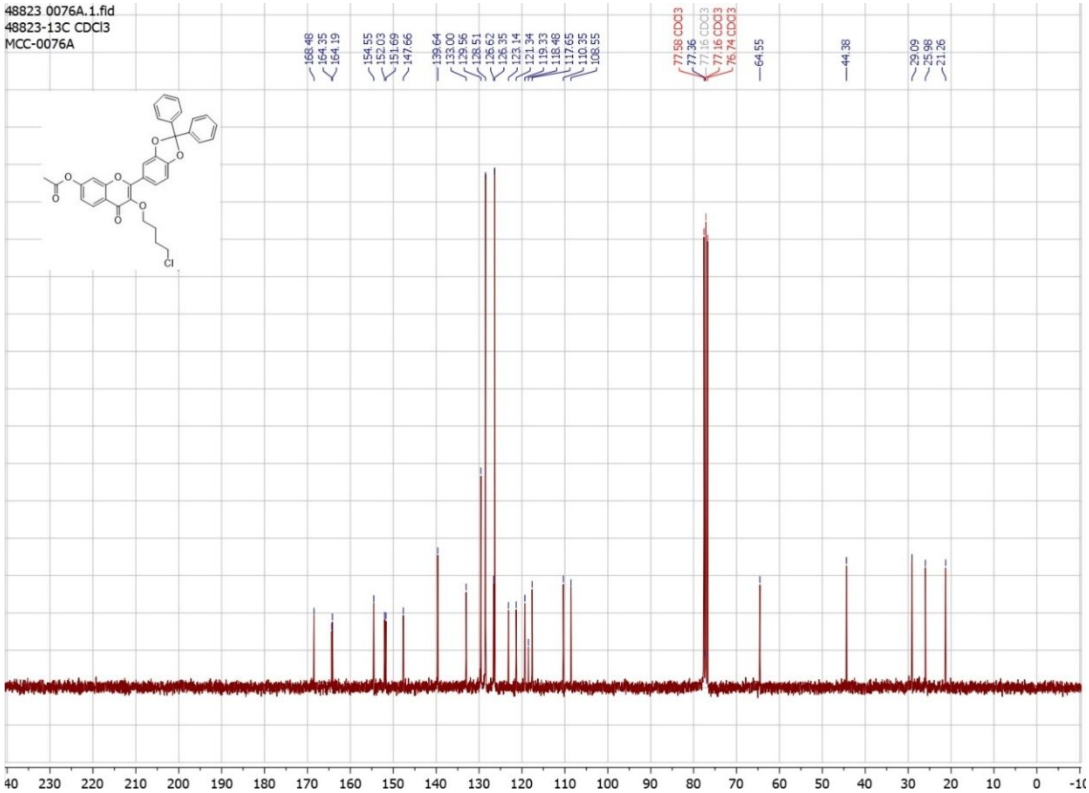

Figure S6. ^{13}C NMR (75 MHz, $CDCl_3$) of 3-(4-chlorobutoxy)-2-(2,2-diphenylbenzo[*d*][1,3]dioxol-5-yl)-4-oxo-4H-chromen-7-yl acetate.

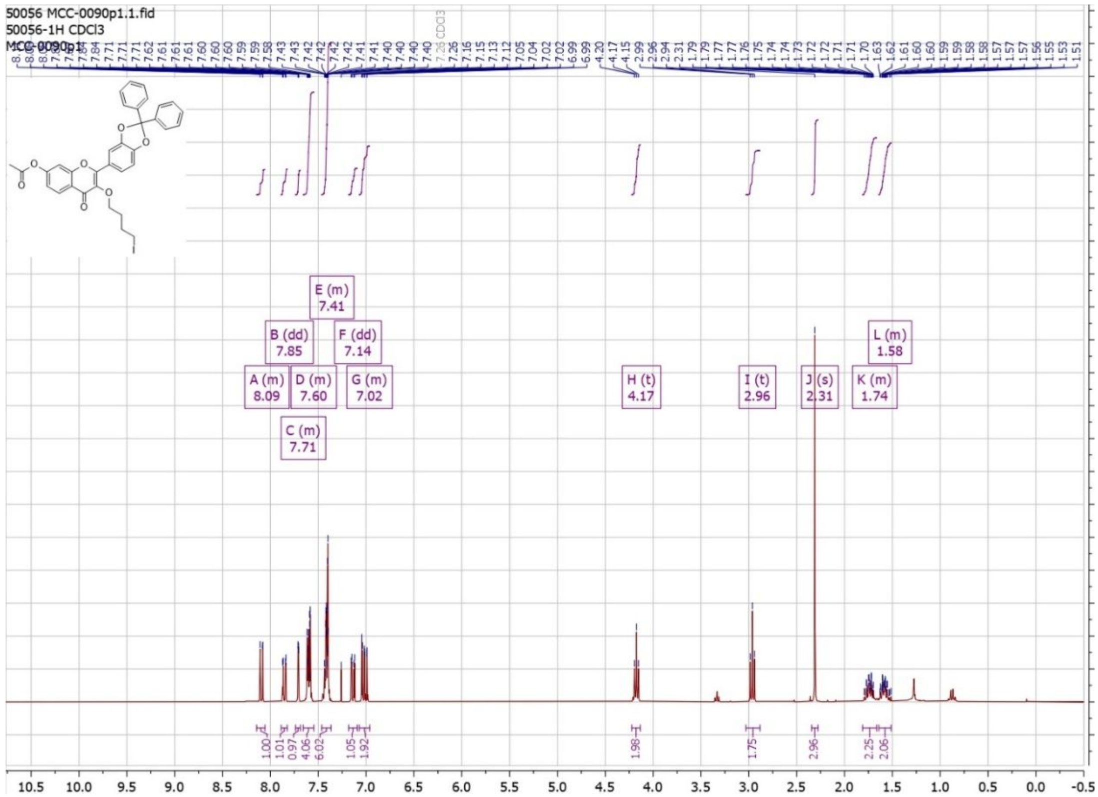

Figure S7. ^{1}H NMR (300 MHz, $CDCl_3$) of 2-(2,2-diphenylbenzo[*d*][1,3]dioxol-5-yl)-3-(4-iodobutoxy)-4-oxo-4H-chromen-7-yl acetate.

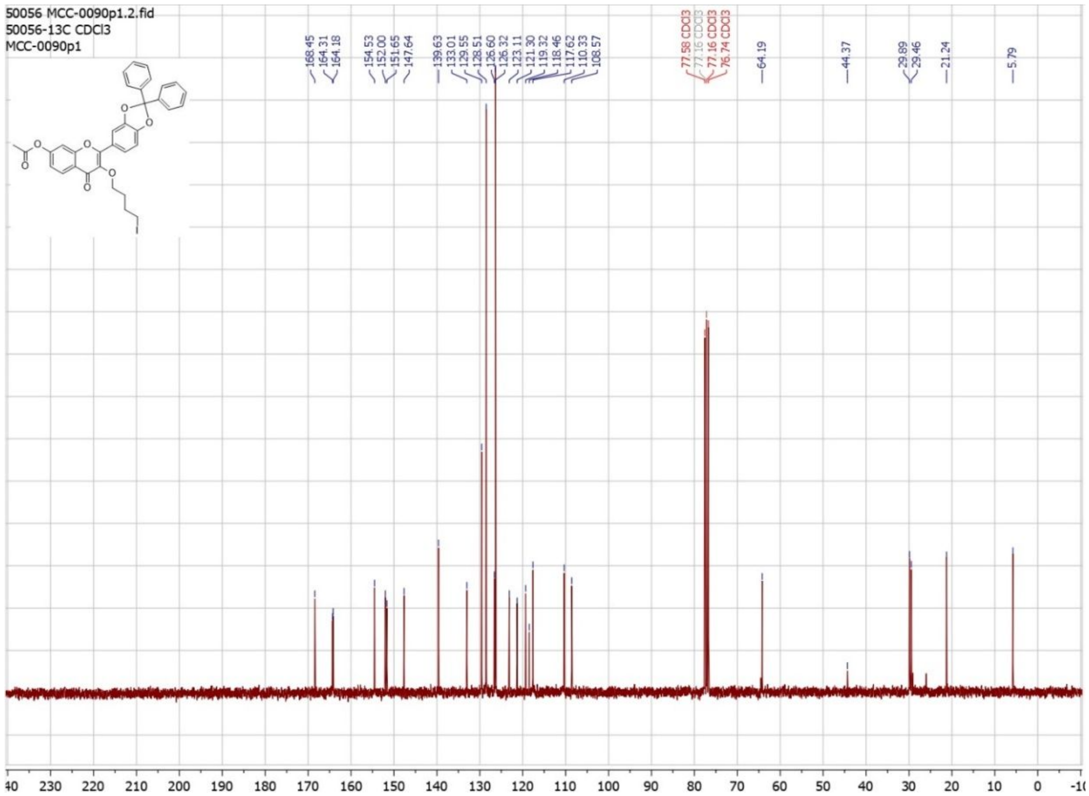

Figure S8. ^{13}C NMR (75 MHz, $CDCl_3$) of 2-(2,2-diphenylbenzo[*d*][1,3]dioxol-5-yl)-3-(4-iodobutoxy)-4-oxo-4H-chromen-7-yl acetate.

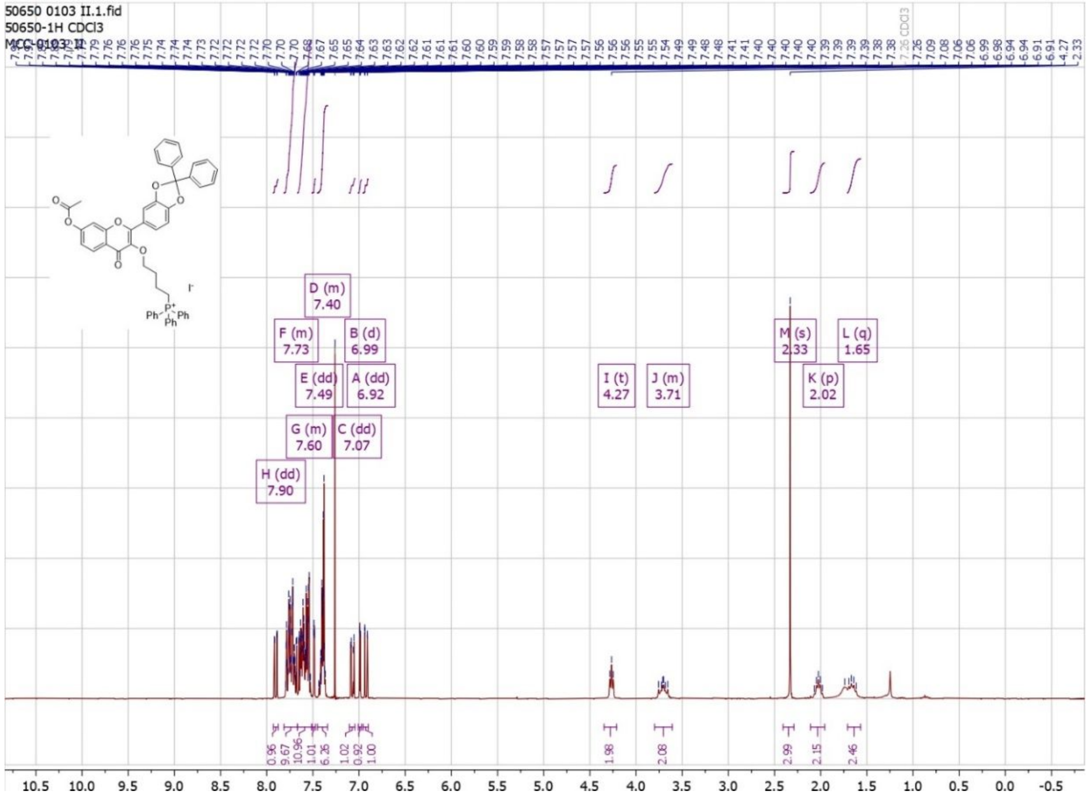

Figure S9. ^1H NMR (300 MHz, $CDCl_3$) of mF3 (4-((7-acetoxy-2-(2,2-diphenylbenzo[*d*][1,3]dioxol-5-yl)-4-oxo-4H-chromen-3-yl)oxy)butyl)triphenylphosphonium iodide.

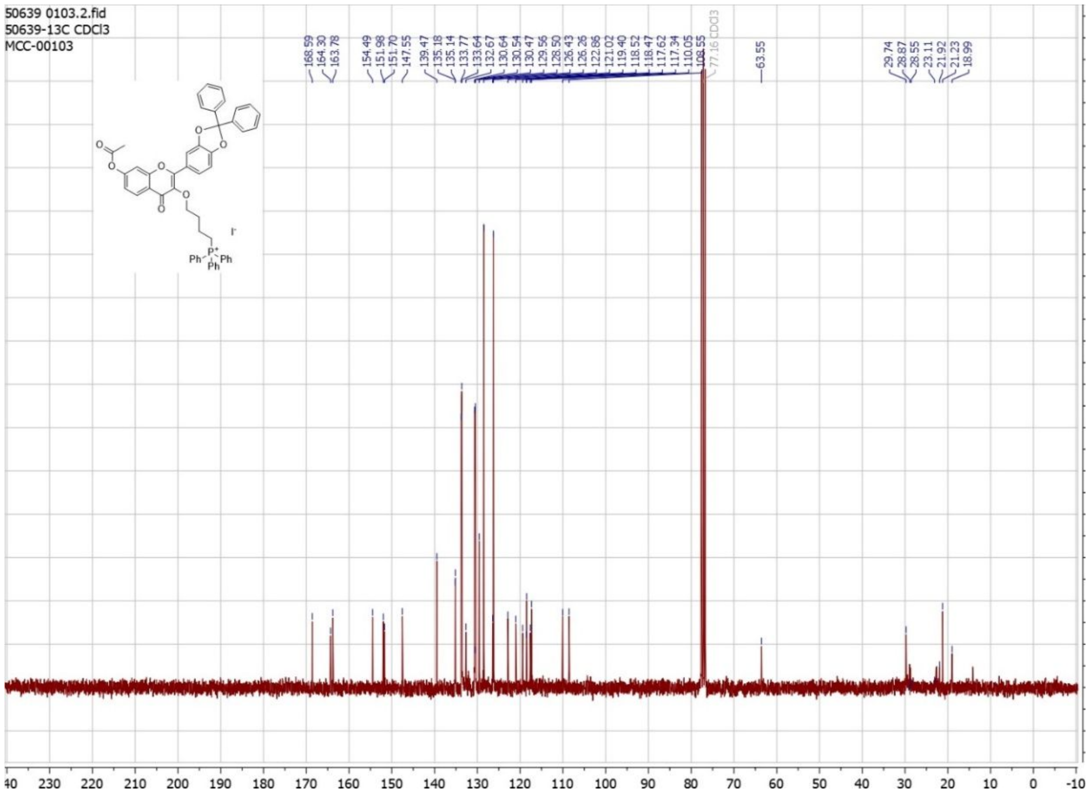

Figure S10. ^{13}C NMR (75 MHz, $CDCl_3$) of mF3 (4-((7-acetoxy-2-(2,2-diphenylbenzo[*d*][1,3]dioxol-5-yl)-4-oxo-4H-chromen-3-yl)oxy)butyl)triphenylphosphonium iodide.

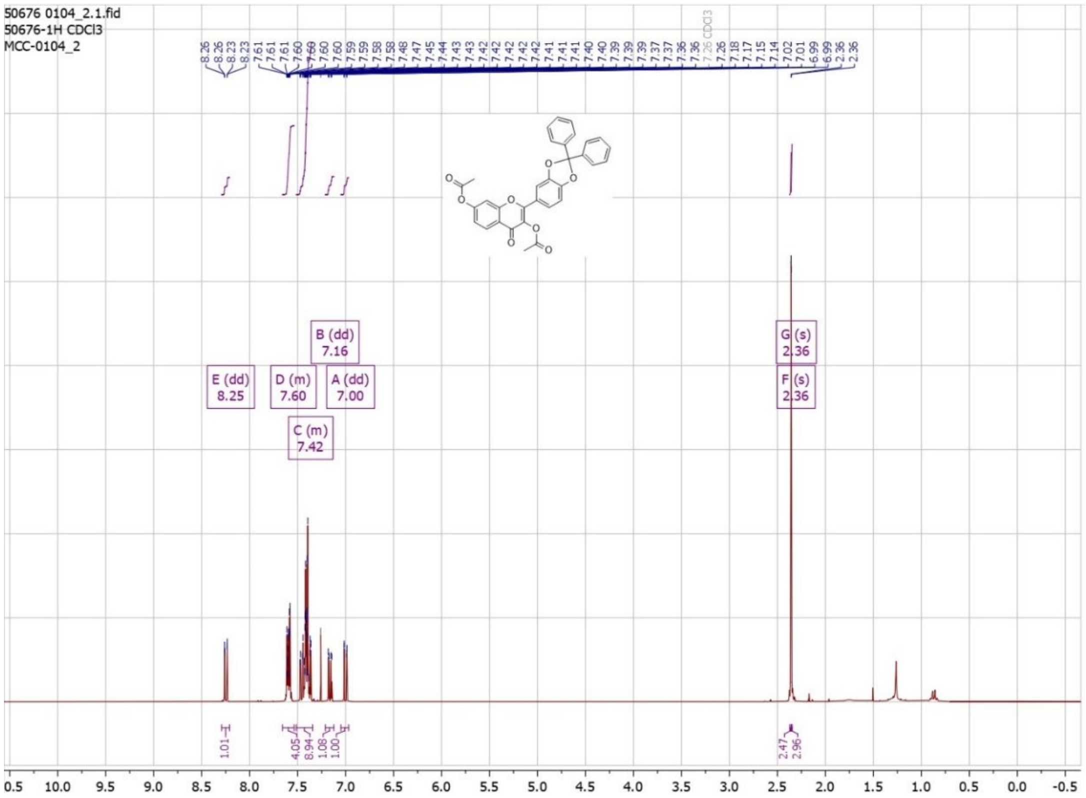

Figure S11. ^{1}H NMR (300 MHz, $CDCl_3$) of 2-(2,2-diphenylbenzo[*d*][1,3]dioxol-5-yl)-4-oxo-4H-chromene-3,7-diyl diacetate.

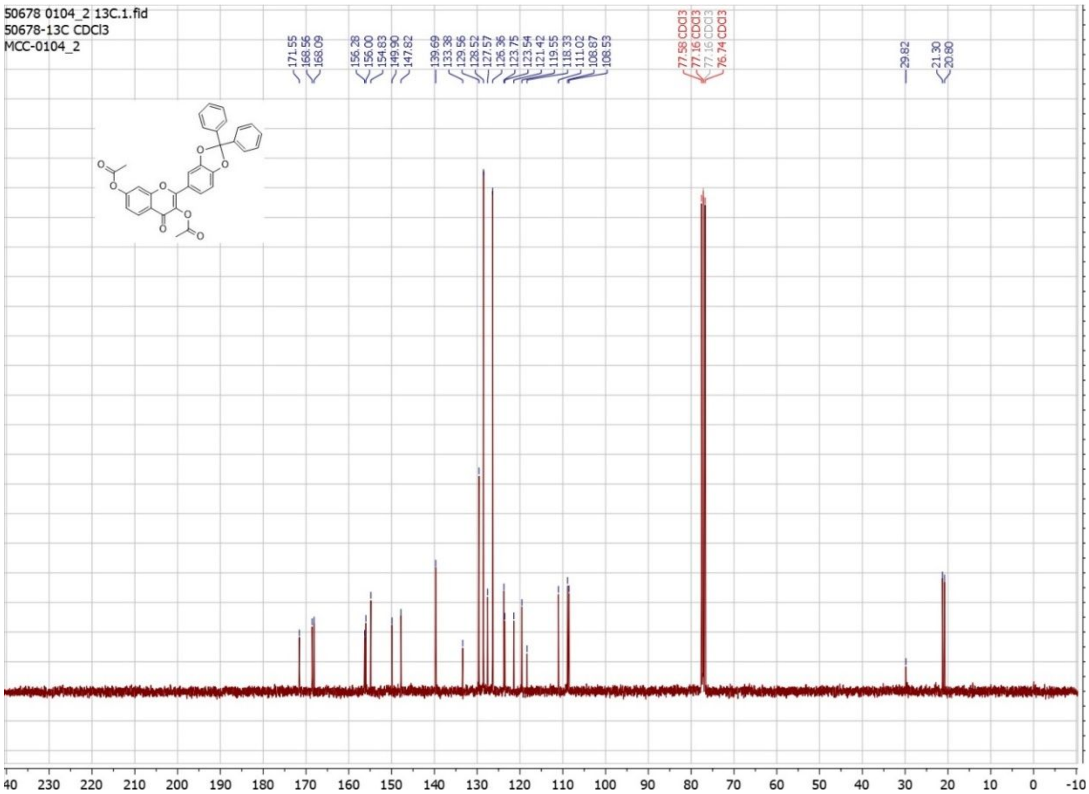

Figure S12. ^{13}C NMR (75 MHz, $CDCl_3$) of 2-(2,2-diphenylbenzo[*d*][1,3]dioxol-5-yl)-4-oxo-4H-chromene-3,7-diyl diacetate.

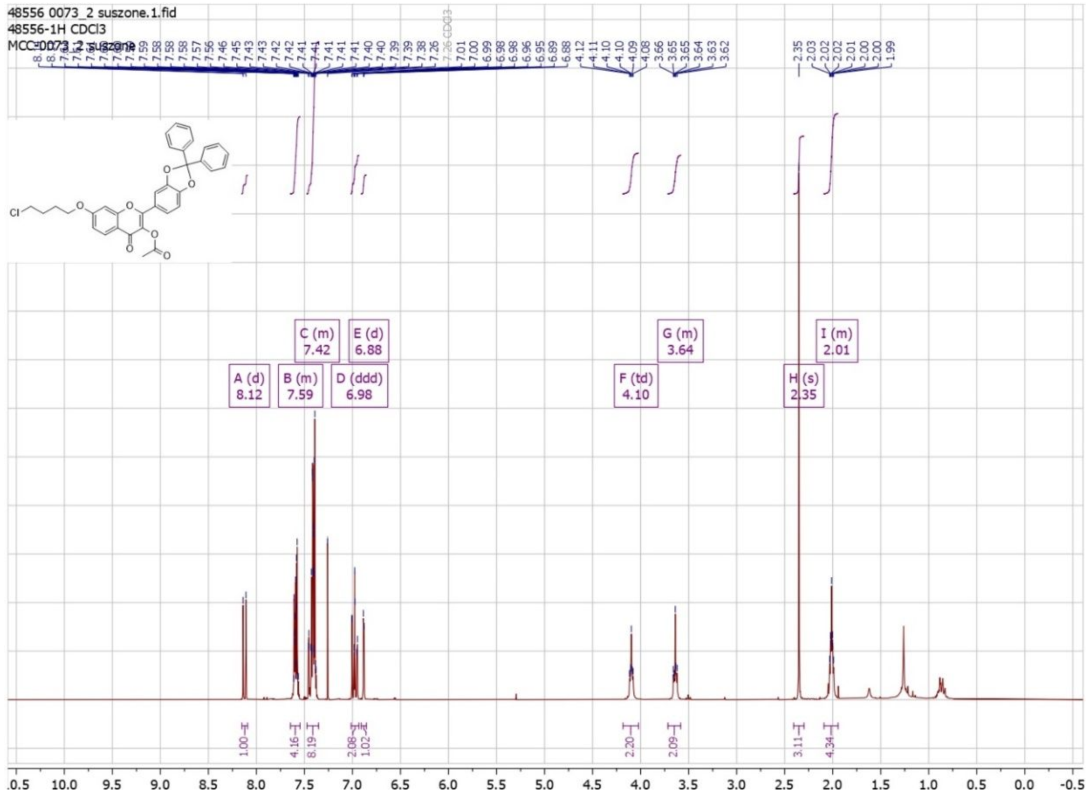

Figure S13. ^{1}H NMR (300 MHz, $CDCl_3$) of 7-(4-chlorobutoxy)-2-(2,2-diphenylbenzo[*d*][1,3]dioxol-5-yl)-4-oxo-4H-chromen-3-yl acetate.

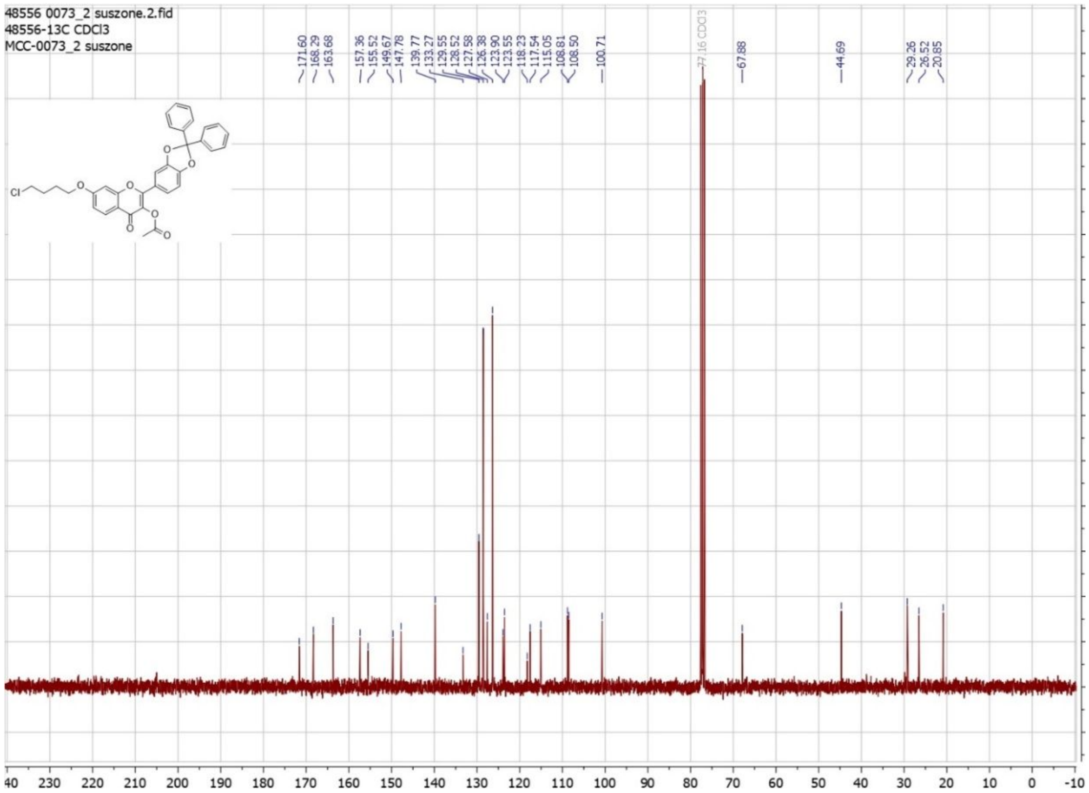

Figure S14. ^{13}C NMR (75 MHz, $CDCl_3$) of 7-(4-chlorobutoxy)-2-(2,2-diphenylbenzo[*d*][1,3]dioxol-5-yl)-4-oxo-4H-chromen-3-yl acetate.

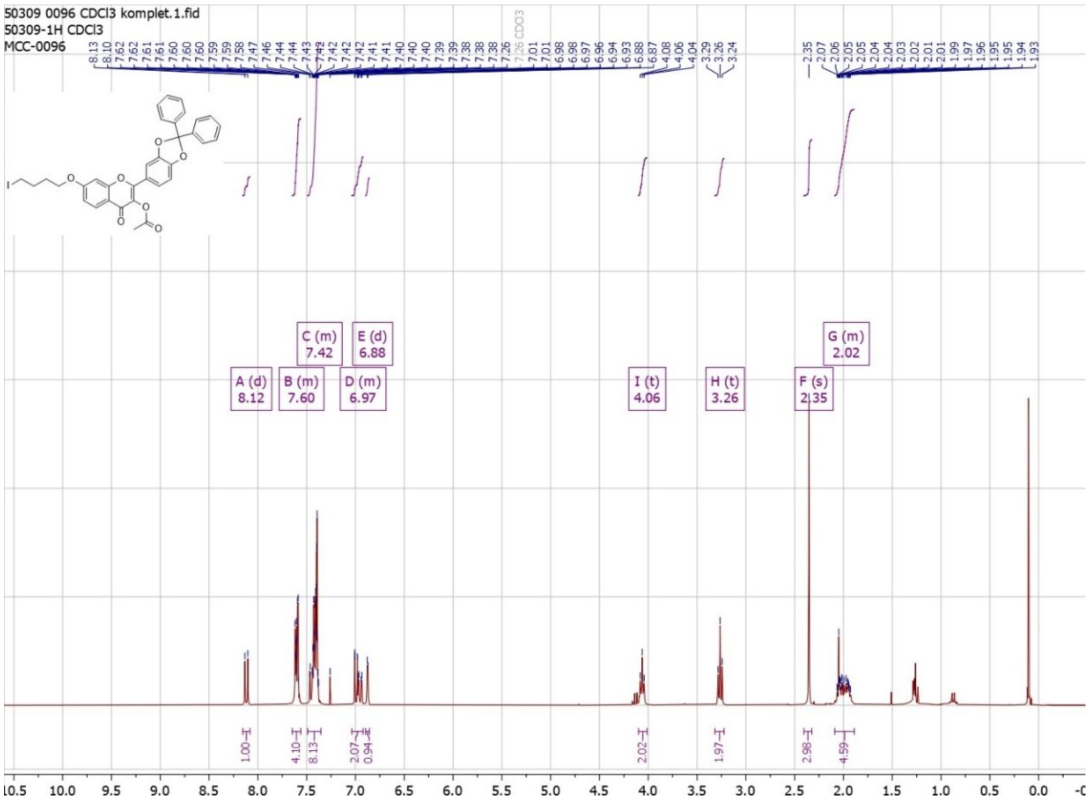

Figure S15. ^{1}H NMR (300 MHz, $CDCl_3$) of 2-(2,2-diphenylbenzo[*d*][1,3]dioxol-5-yl)-7-(4-iodobutoxy)-4-oxo-4H-chromen-3-yl acetate.

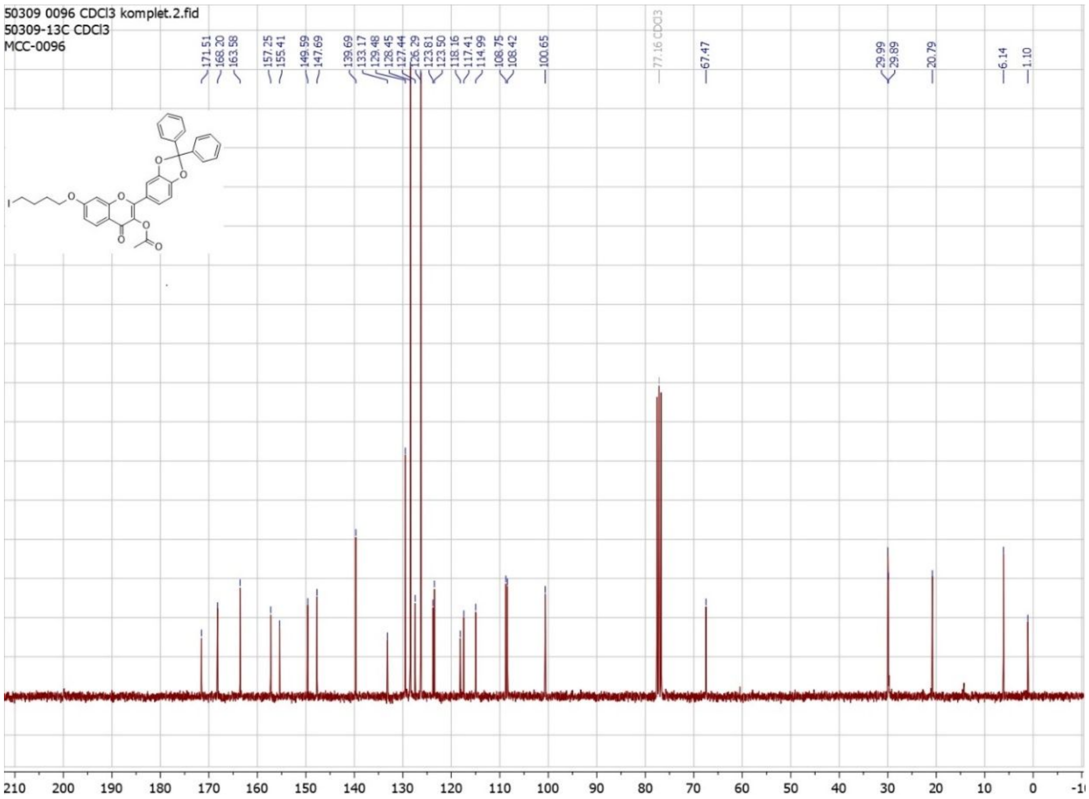

Figure S16. ^{13}C NMR (75 MHz, $CDCl_3$) of 2-(2,2-diphenylbenzo[*d*][1,3]dioxol-5-yl)-7-(4-iodobutoxy)-4-oxo-4H-chromen-3-yl acetate.

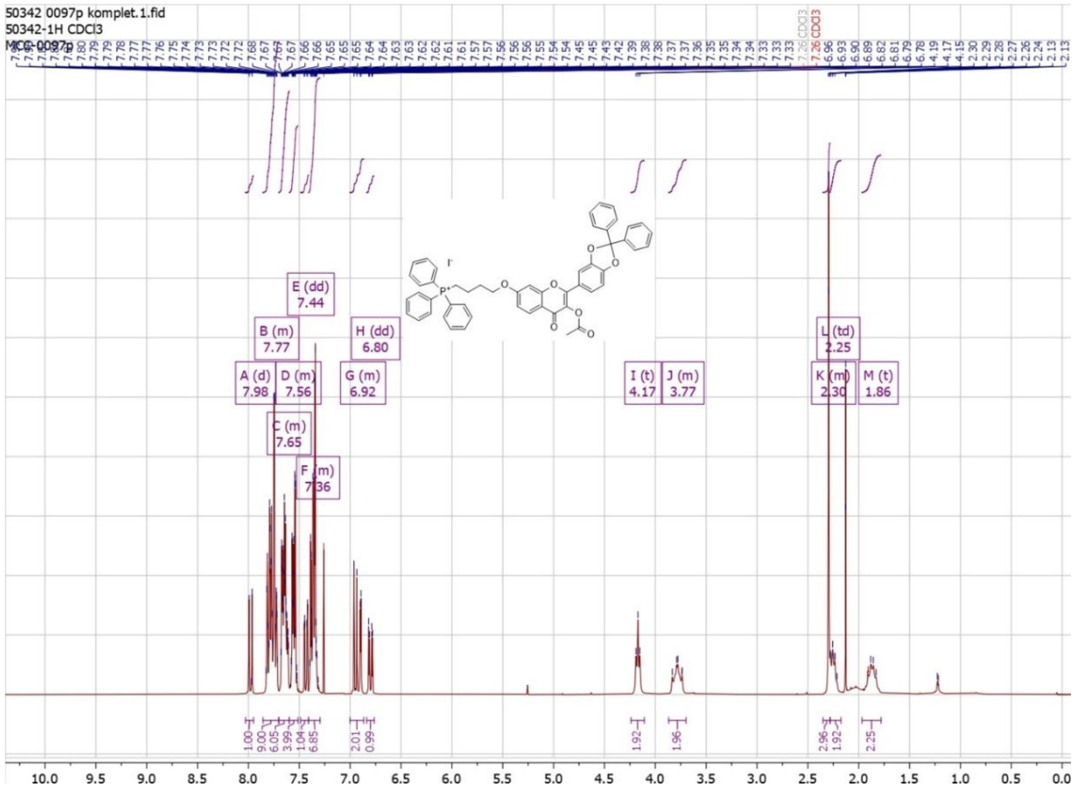

Figure S17. ^{1}H NMR (300 MHz, $CDCl_3$) of mF7 (4-((3,5-diacetoxy-2-(2,2-diphenylbenzo[*d*][1,3]dioxol-5-yl)-4-oxo-4H-chromen-7-yl)oxy)butyl)triphenylphosphonium iodide.

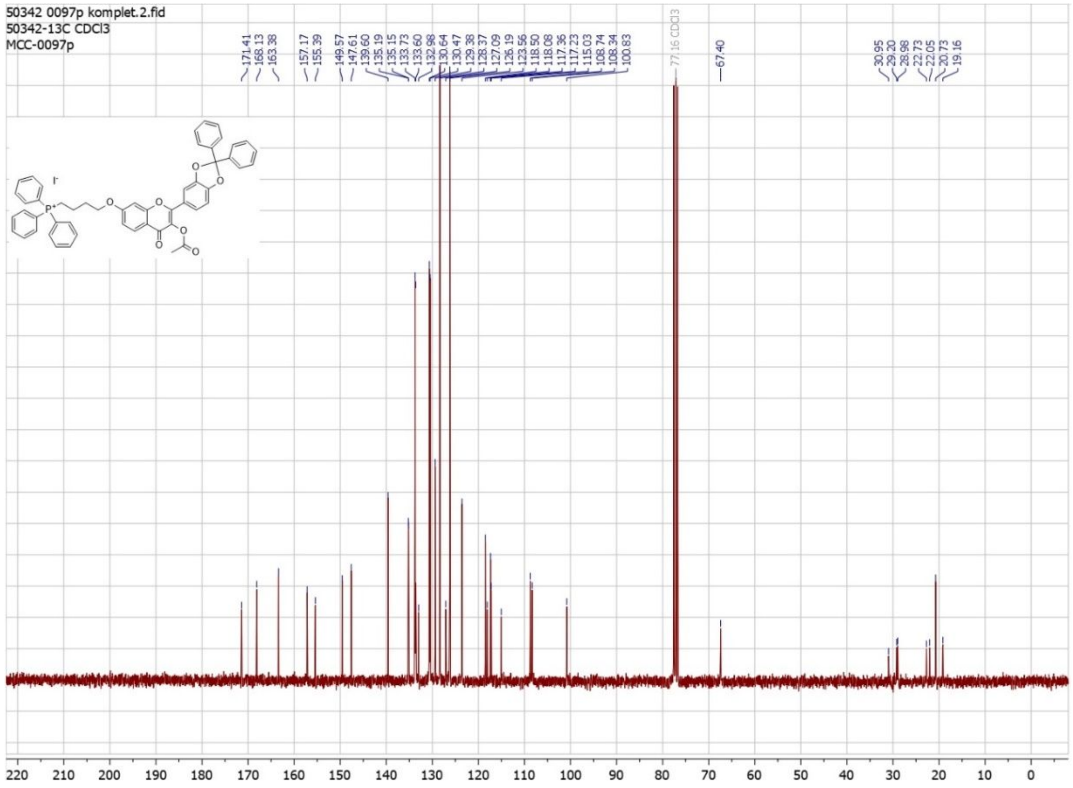

Figure S18. ^{13}C NMR (75 MHz, $CDCl_3$) of mF7 (4-((3,5-diacetoxy-2-(2,2-diphenylbenzo[*d*][1,3]dioxol-5-yl)-4-oxo-4H-chromen-7-yl)oxy)butyl)triphenylphosphonium iodide.

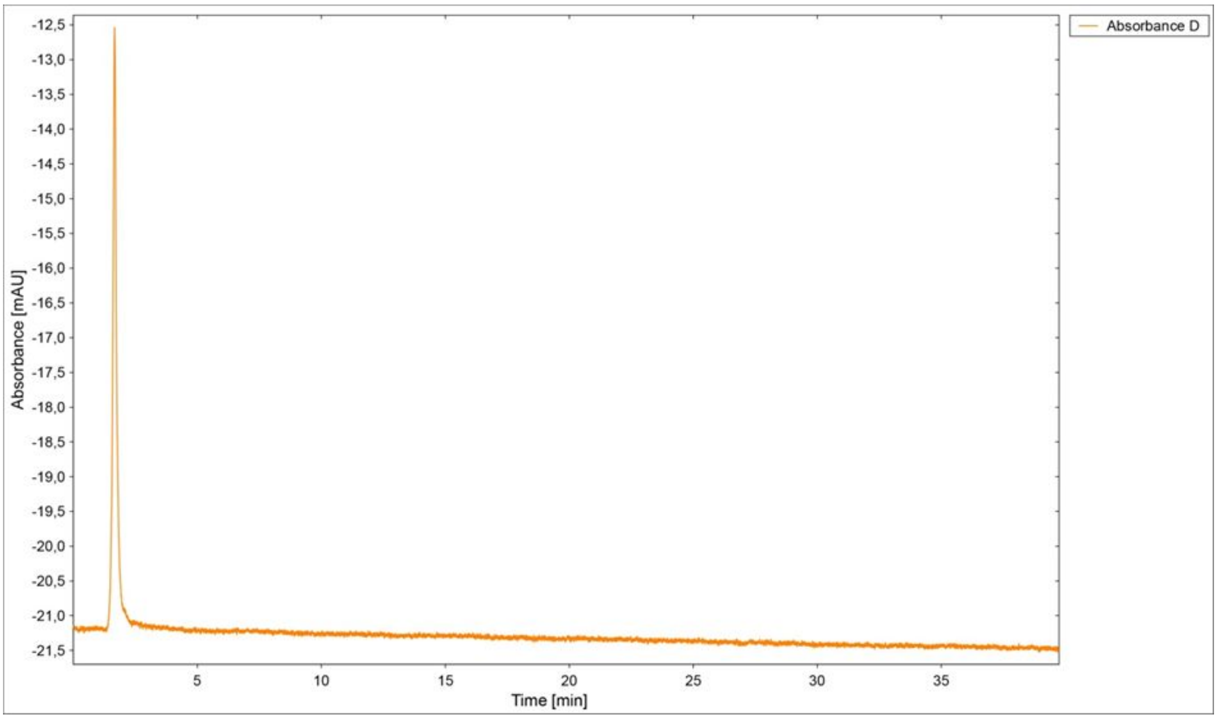

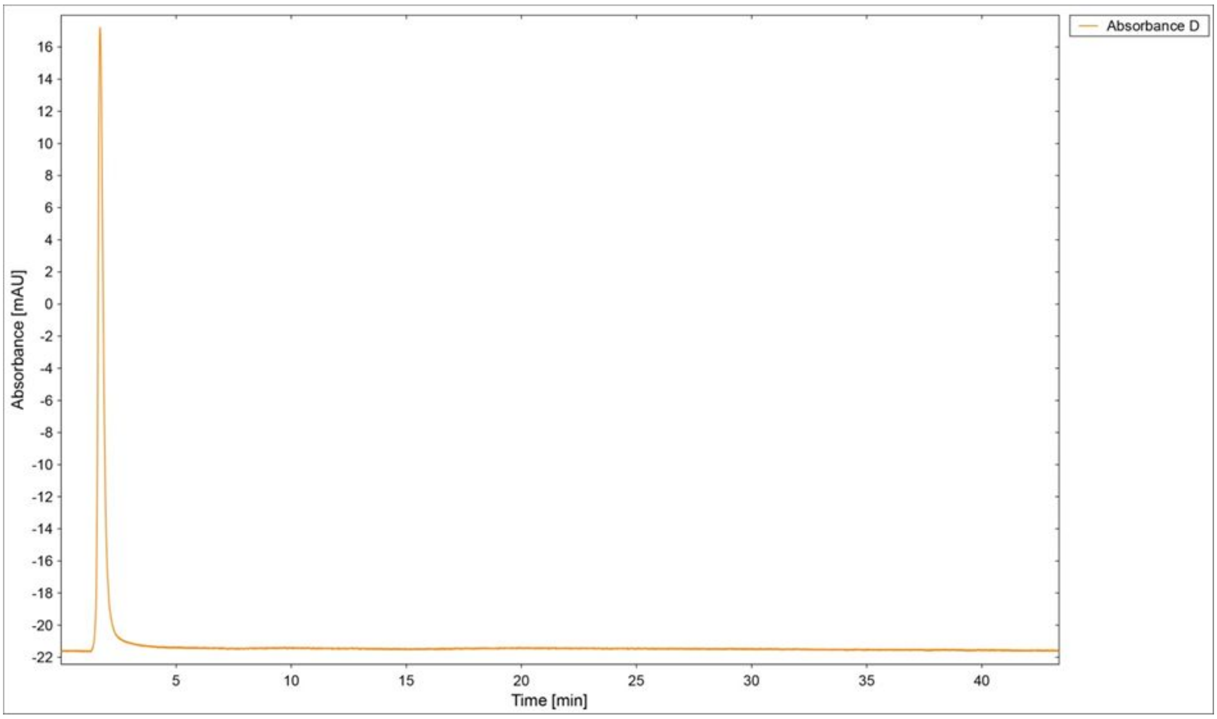

Figure S19. Normal phase isocratic HPLC profiles for final compounds: mF3 (upper panel, Rt = 1.72 min) and mF7 (lower panel, RT = 1.69 min). In both analyzed samples, the level of impurities is below 0.1% (no impurity peaks detected). Conditions: JAI LaboACE LC-9160NEXT II, column Phenomenex Phenogel 50A 7.8mm x 300mm, detector UV-Vis 4CH NEXT, wavelength 365 nm, eluent THF (6 ml/min).

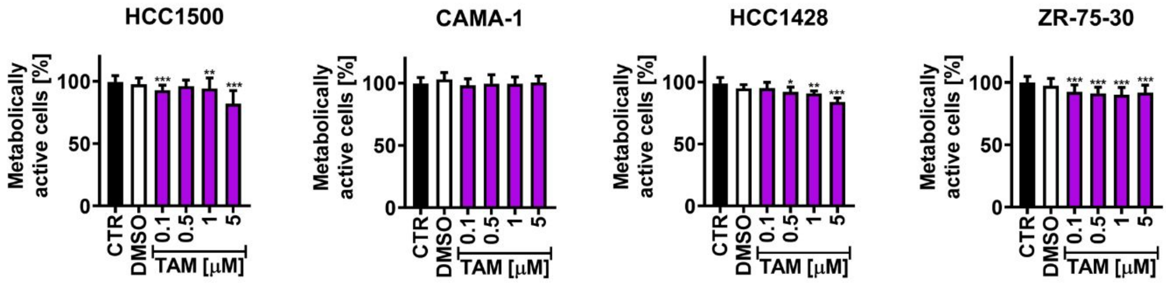

Figure S20. Changes in the metabolic activity of ER-positive breast cancer cells (HCC1500, CAMA-1, HCC1428, and ZR-75-30) upon stimulation with tamoxifen (TAM). Cells were treated with TAM (0.1, 0.5, 1, and 5 μM) for 24 h and metabolic activity was assessed using MTT test. The effect of solvent used (DMSO) is also presented. Bars indicate SD, n = 3, $^{***}p<0.001$, $^{**}p<0.01$, $^{*}p<0.05$ compared to corresponding untreated control (CTR) (ANOVA and Dunnett's a posteriori test).

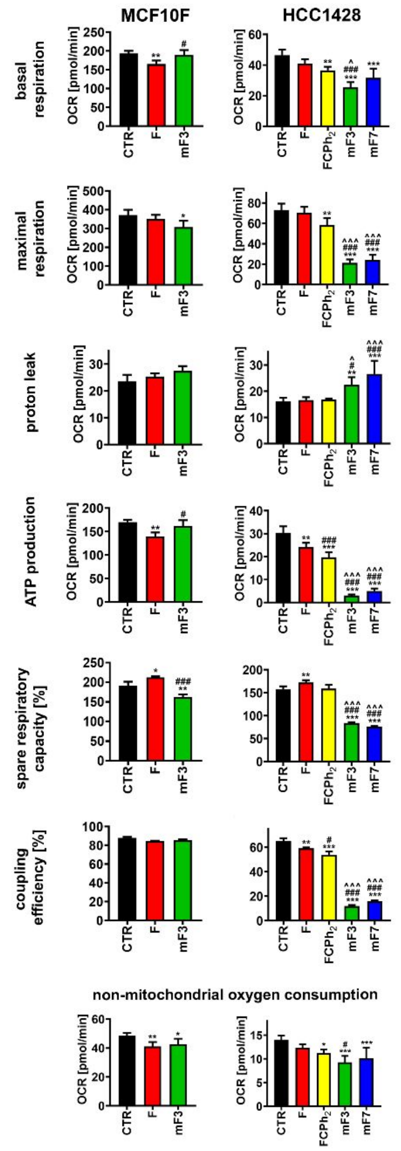

Figure S21. Fisetin derivative-mediated changes in mitochondrial parameters in proliferating non-cancerous MCF10F cells and HCC1428 breast cancer cells. Cells were treated with 5 µM fisetin (F) or fisetin derivatives ($FCPh_2$, mF3, and mF7) for 6 h. Mitochondrial function was assayed as real-time measurements of mitochondrial oxidative phosphorylation (OXPHOS) as selected OCR parameters (pmol/min or %), namely basal respiration, ATP production, and proton leak (stimulation with oligomycin), maximal respiration (stimulation with the uncoupler FCCP) and spare respiratory capacity (stimulation with rotenone and antimycin A). Uncoupling efficiency [%] and non-mitochondrial oxygen consumption [pmol/min] are also

presented. Bars indicate SD, n = 3, $^{***}p<0.001$, $^{**}p<0.01$, $^{*}p<0.05$ compared to corresponding untreated control (CTR) (ANOVA and Dunnett's a posteriori test), $^{\#\#\#}p<0.001$, $^{\#}p<0.05$ compared to fisetin treatment (F) (ANOVA and Tukey's a posteriori test), $^{\wedge\wedge\wedge}p<0.001$, $^{\wedge}p<0.05$ compared to $FCPh_2$ treatment (ANOVA and Tukey's a posteriori test).

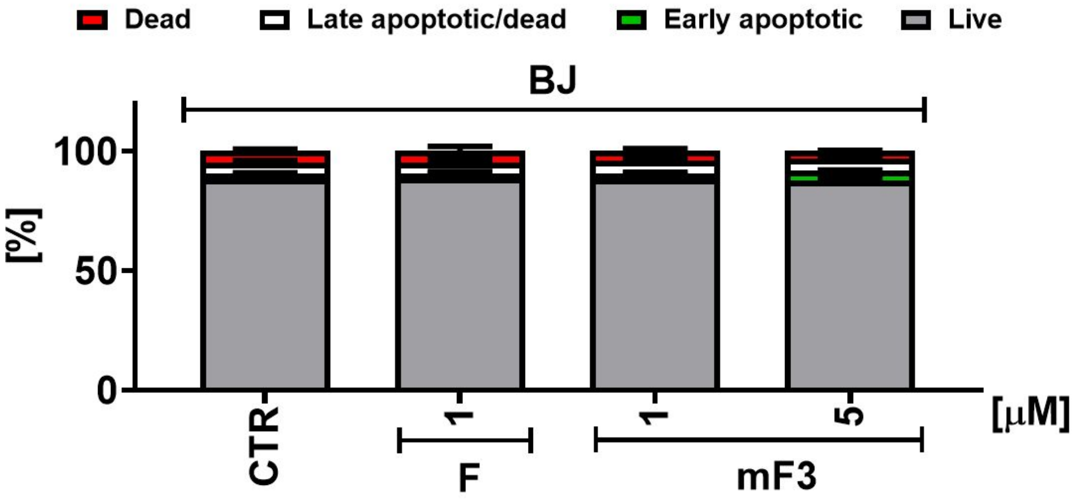

Figure S22. Mito-fisetin (mF3)-mediated apoptosis in BJ human fibroblasts. BJ cells were treated with fisetin (F, 1 μM) and mito-fisetin (mF3, 1 and 5 μM) for 24 h. Phosphatidylserine externalization as a marker of apoptosis was analyzed using Annexin V staining and flow cytometry. Bars indicate SD, n = 3. CTR, control conditions.

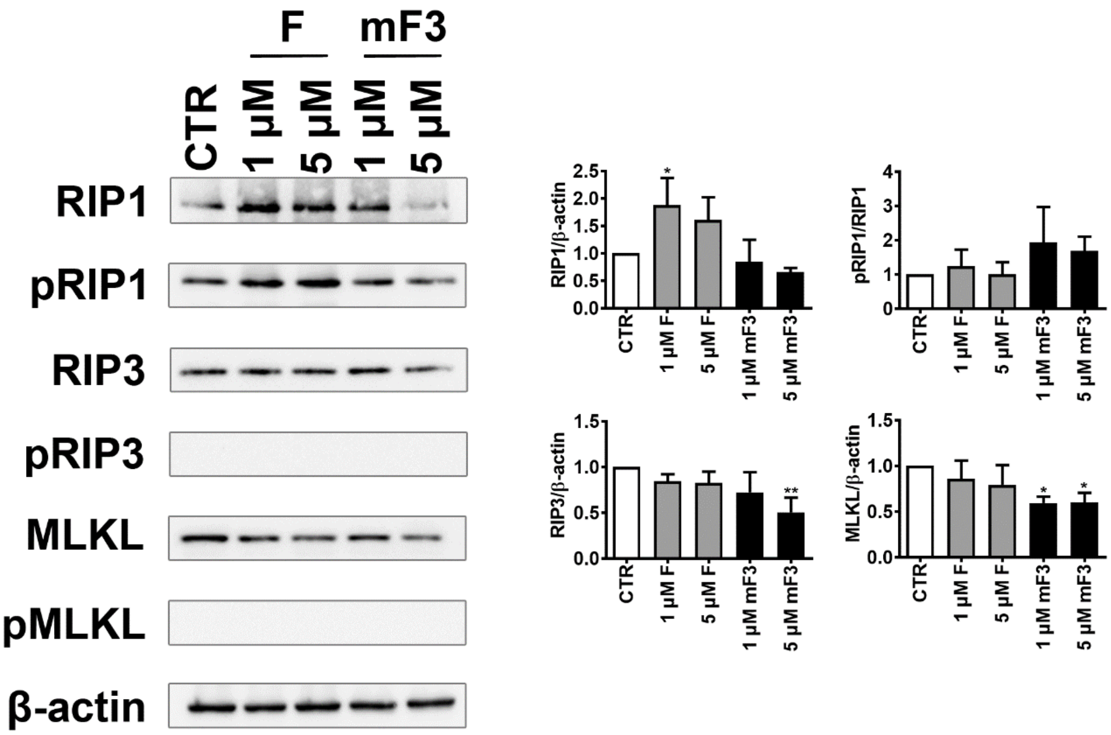

Figure S23. The effect of mito-fisetin (mF3) on the levels of key markers of necroptotic cell death in HCC1428 breast cancer cells. Cells were treated with 1 or 5 µM fisetin (F) or 1 or 5 µM mito-fisetin (mF3) for 24 h. The levels of RIP1, phospho-RIP1 (pRIP1), RIP3, phospho-RIP3 (pRIP3), MLKL, and phospho-MLKL (pMLKL) were evaluated using western blotting (**left**, representative blots; **right**, quantitative analysis). Data were normalized to a loading control (β-actin). For the analysis of phosphorylation status of RIP1, the ratio of phospho-RIP1 to RIP1 was calculated. As no phosphorylated signals of RIP3 and MLKL were detected (**left**), similar analysis was not possible to assess the ratio of phospho-RIP3 to RIP3 and phospho-MLKL to MLKL, respectively. Bars indicate SD, n = 3, $^{**}p<0.01$, $^{*}p<0.05$ compared to corresponding untreated control (CTR) (ANOVA and Dunnett's a posteriori test).

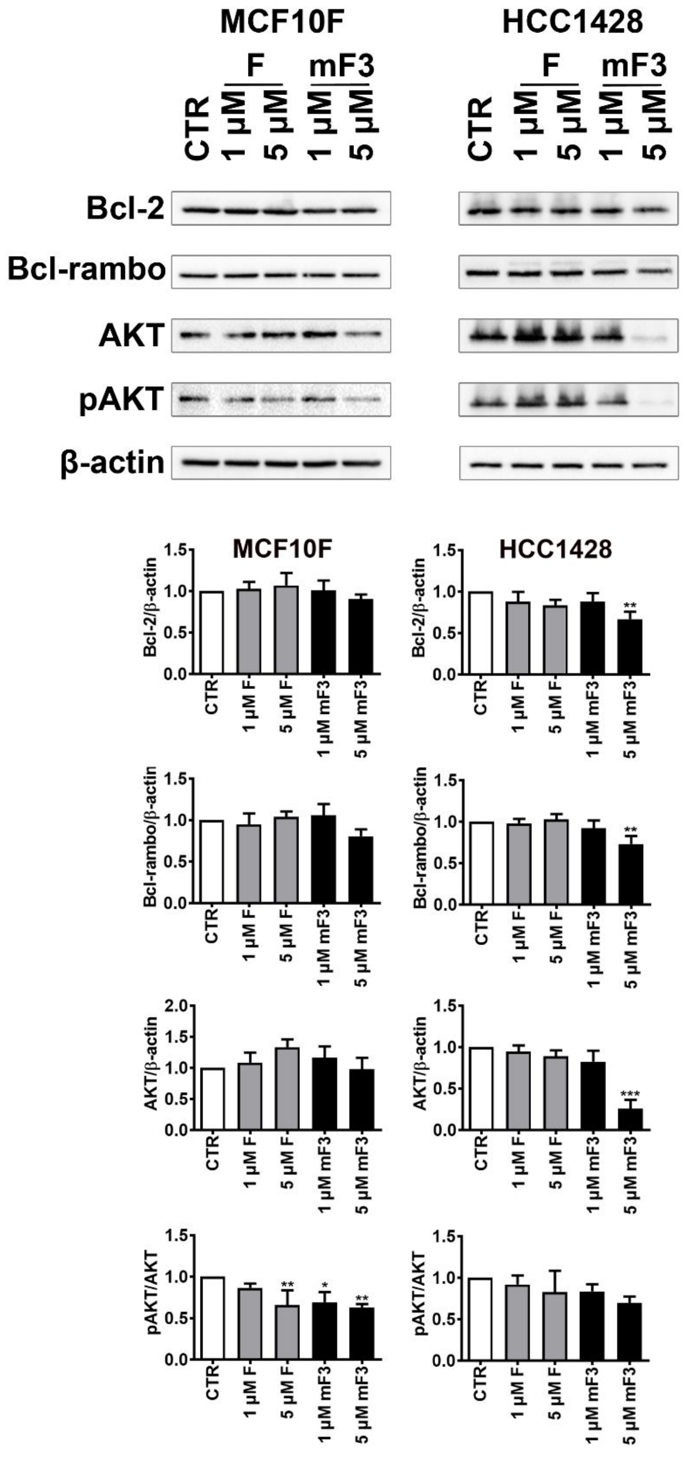

Figure S24. Western blot-based analysis of the levels of Bcl-2, Bcl-rambo, AKT, and phospho-AKT in mF3-treated proliferating normal MCF10F cells and HCC1428 breast cancer cells. Cells were treated with 1 or 5 μM fisetin (F) or 1 or 5 μM mito-fisetin (mF3) for 24 h. Data were normalized to a loading control (β-actin) (**bottom**). For the analysis of

phosphorylation status of AKT, the ratio of phospho-AKT to AKT was calculated. Bars indicate SD, n = 3, $^{***}p<0.001$, $^{**}p<0.01$, $^{*}p<0.05$ compared to corresponding untreated control (CTR) (ANOVA and Dunnett's a posteriori test). Representative blots are also shown (**top**).
